# Operando Characterization of Fe in Doped Ni_*x*_(Fe_1–*x*_)O_*y*_H_*z*_ Catalysts for Electrochemical Oxygen Evolution

**DOI:** 10.1021/jacs.4c13417

**Published:** 2025-01-25

**Authors:** Joakim Halldin Stenlid, Mikaela Görlin, Oscar Diaz-Morales, Bernadette Davies, Vladimir Grigorev, David Degerman, Aleksandr Kalinko, Mia Börner, Mikhail Shipilin, Matthias Bauer, Alessandro Gallo, Frank Abild-Pedersen, Michal Bajdich, Anders Nilsson, Sergey Koroidov

**Affiliations:** † Department of Physics, Alba Nova Research Center, Stockholm University, Stockholm SE-106 91 Sweden; ‡ SUNCAT Center for Interface Science and Catalysis, SLAC National Accelerator Laboratory, 2575 Sandhill Road, Menlo Park, California 94025, United States; § SUNCAT Center for Interface Science and Catalysis, Department of Engineering, Stanford University, 443 Via Ortega, Stanford, California 94305, United States; ∥ Department of Chemistry, Ångström Laboratory, Uppsala University, Uppsala SE-751 21, Sweden; ⊥ Holst Centre, Netherlands Organisation for Applied Scientific Research, HighTech Campus 31, Eindhoven, 5656, the Netherlands; # Department of Chemistry and Center for Sustainable Systems Design (CSSD), University of Paderborn, Warburger Strasse 100, Paderborn D-33098, Germany; ∇ Deutsches Elektronen-Synchrotron DESY, Notkestraße 85, Hamburg D-22607, Germany; ○ Sila Nanotechnologies, 2470 Mariner Square Loop, Alameda, California 94501, United States; ◆ Wallenberg Initiative Materials Science for Sustainability (WISE), Department of Physics, Stockholm University, Stockholm SE-106 91, Sweden

## Abstract

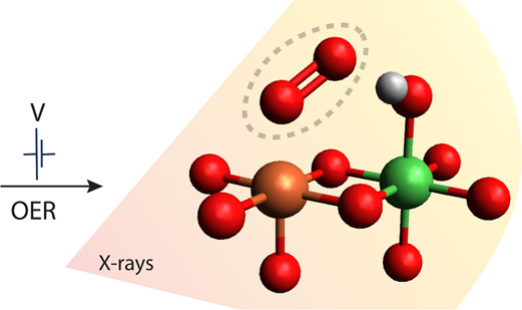

Iron-doped nickel oxyhydroxides, Ni_*x*_(Fe_1–*x*_)O_*y*_H_*z*_, are among the most promising oxygen evolution reaction (OER) electrocatalysts in alkaline environments. Although iron (Fe) significantly enhances the catalytic activity, there is still no clear consensus on whether Fe directly participates in the reaction or merely acts as a promoter. To elucidate the Fe’s role, we performed *operando* X-ray spectroscopy studies supported by DFT on Ni_*x*_(Fe_1–*x*_)O_*y*_H_*z*_ electrocatalysts. We probed the reversible changes in the structure and electronic character of Ni_*x*_(Fe_1–*x*_)O_*y*_H_*z*_ as the electrode potential is cycled between the resting (here at 1.10 *V*_RHE_) and operational states (1.66 *V*_RHE_). DFT calculations and XAS simulations on a library of Fe structures in various NiO_*y*_H_*z*_ environments are in favor of a distorted local octahedral Fe(III)O_3_(OH)_3_ configuration at the resting state with the NiO_*y*_H_*z*_ scaffold going from α-Ni(OH)_2_ to γ-NiOOH as the potential is increased. Under catalytic conditions, EXAFS and HERFD spectra reveal changes in *p*-*d* mixing (covalency) relative to the resting state between O/OH ligands and Fe leading to a shift from octahedral to square pyramidal coordination at the Fe site. XES measurements and theoretical simulations further support that the Fe equilibrium structure remains in a formal Fe(III) state under both resting and operational conditions. These spectral changes are attributed to potential dependent structural rearrangements around Fe. The results suggest that ligand dissociation leads to the *C*_4v_ symmetry as the most stable intermediate of the Fe during OER. This implies that Fe has a weakly coordinated or easily dissociable ligand that could serve to coordinate the O–O bond formation and, tentatively, play an active role in the Ni_*x*_(Fe_1–*x*_)O_*y*_H_*z*_ electrocatalyst.

## Introduction

Electrochemical water splitting (2H_2_O → 2H_2_ + O_2_) by renewable energy sources offers an attractive route toward carbon-free hydrogen (H_2_) production for application in, e.g., the transportation and energy sectors, as well as the chemical industry.^1,2^ A remaining barrier to large-scale production of affordable clean hydrogen is that the anodic oxygen evolution reaction (OER) requires rare and expensive noble metal catalysts to overcome the slow kinetics resulting in increased costs for energy-efficient catalysts.^3^ Under alkaline conditions, iron-doped nickel (oxy)hydroxides, Ni_*x*_(Fe_1–*x*_)O_*y*_H_*z*_, are among the most promising emerging OER electrocatalysts with excellent performance^4−8^ compared to other nonprecious metal-based catalysts. Fe is essential for the catalytic activity of the material, but its exact role and character during operation remains elusive.

In general, nickel (oxy)hydroxides belong to a class of two-dimensional dimensional hydrotalcite-like clay materials known as layered double hydroxides.^9^ These have a flexible chemical composition, with or without an intercalating layer of aqueous cations or anions, and where the structure can harbor a broad range of metal centers of different oxidation states from M(II) to M(IV).^10^ Fe-free, undoped NiO_*y*_H_*z*_ is a rather poor OER catalyst. However, incorporation of Fe ions to form Ni_*x*_(Fe_1–*x*_)O_*y*_H_*z*_ catalysts yields one of the most active alkaline OER catalysts reported based on nonprecious elements, with optimal performance found for a 20–40% Fe content.^4,5,8^ Catalytic OER current density of 10 mA cm^–2^ can be reached at ∼0.3 V overpotential in pH 13–14 in KOH electrolyte,^7,8^ although a decrease in overpotential and improvements in the long term stability are still needed for industrial feasibility.

A key to such further advancements is the understanding how Fe enhances the OER activity of the parent catalyst. This is a highly active research field with sometimes seemingly conflicting findings.^11−17^ Various factors have been examined and advocated, including Fe as the active center,^18,19^ Fe as a promotor of Ni active sites,^20^ and a bifunctional active site comprising both Fe and Ni.^21^ Computational studies investigating the roles of Ni versus Fe, as well as the significance of defects at edge, corner, and bulk active sites, have progressively provided more detailed mechanistic insights.^14,16,18,22−29^ Yet, a consensus about the exact role and character of Fe under operation still eludes the field. The disagreement over fundamental aspects, such as the oxidation state or local structure of the Fe site at catalytic conditions, highlights the challenges in probing Fe during its operational state. Numerous contradictory studies provide evidence that Fe ions either are electrochemically oxidized to Fe(IV),^30^ Fe(>IV),^31^ or remain Fe(III)^18^ under catalytic conditions. Structurally, Fe has been proposed to remain in the octahedral (*O*_h_) coordination of the NiO_*y*_H_*z*_ scaffold,^32^ or to undergo reconstruction to square pyramidal (*C*_4v_), planar, or tetrahedral (*T*_d_) coordination structure upon the loss of one or two ligands forming Fe(V) and Fe(VI) respectively.^31^ Fe has also been proposed to be a dynamic center, switching between a dissolved and immobilized state.^19^ In addition, it remains unclear whether Fe is perfectly integrated into the NiO_*y*_H_*z*_ or forms local FeO_*y*_H_*z*_ patches with high Fe concentration.^18^ The available literature on these proposals provides little conclusive evidence in either direction.

*In situ* experimentation has significantly been aided by improvements in instrumentation, sample environment cells, and light sources over the last decades. This has made it possible to employ X-ray spectroscopy techniques for probing (electro-)catalysts under operation with a high level of detail.^33−35^ For instance, *K*-edge X-ray absorption spectroscopy (XAS) using high-energy resolution fluorescence detection (HERFD) has been used to reveal the character of Co in the LiCoO_2_ cathode of Li-batteries,^36^ as well as in the alkaline water splitting catalysts for CoOOH,^37^ MnO_*x*_,^38^ IrO_*x*_,^39^ and the character of the Ni site in Ni_*x*_(Fe_1–*x*_)O_*y*_H_*z*_.^18,40^ Extended X-ray absorption fine structure (EXAFS)^18,29,41,42^ and X-ray emission spectroscopy (XES)^43−47^ are alternative techniques, widely used to probe the character of transition metal centers under operation.

Here, we combine various *operando* X-ray spectroscopy techniques, encompassing HERFD, EXAFS, as well as *K*α and *K*β XES, with density functional theory (DFT) simulations, including spectra simulation, to gain knowledge about the Fe-site in the Ni_*x*_(Fe_1–*x*_)O_*y*_H_*z*_ electrocatalyst under OER operating conditions. Our results enable the assignment of characteristic spectral changes with reversible electronic and structural alterations of Fe as the applied potential bias is cycled from a resting (1.10 *V*_RHE_) to an operational (1.66 *V*_RHE_) state of the catalyst material. Whereas transient states of Fe under the catalytic cycle cannot be probed, the results show that the equilibrium structure of Fe remains Fe(III) under catalytic conditions with a *C*_4v_ Fe coordination as a likely key structure in the catalytic OER cycle.

### Conceptual Background of the X-ray Spectroscopy (XAS and XES) Techniques

**Figure 1:**
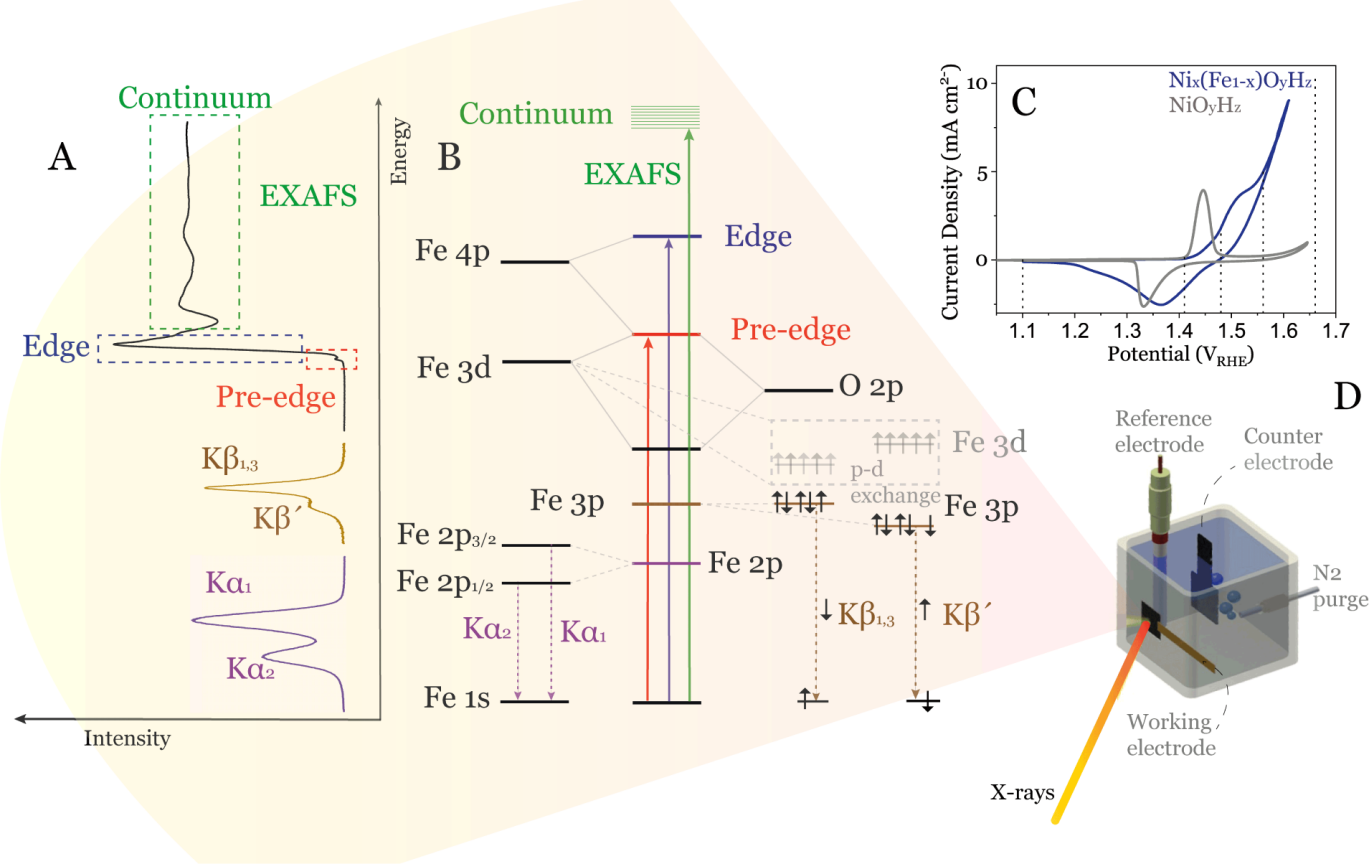
(A) Example Fe K-edge X-ray absorption data with pre-edge, edge, and EXAFS regions and spectral shape of Kβ main and Kα emission lines. (B) Simplified diagram of transitions that contribute to Fe K-edge XAS as well as Fe Kβ and Kα XES. (C) Polarization curve showing electrocatalytic OER performance for Ni_*x*_(Fe_1–*x*_)O_*y*_H_*z*_ sample (blue) compared to NiO_*y*_H_*z*_ (gray). Dotted vertical lines indicate potentials where *operando* X-ray data were collected. (D) Schematic view of the electrochemical cell for *operando* X-ray experiments.

An X-ray absorption spectrum is obtained when a core electron is supplied with sufficient X-ray energy to be excited to the unoccupied states. XAS can be divided into different regions depending on the energy of the receiving states, see Figure 1.^33,34^ Excitation into the lowest lying (valence/conduction band) electronic states is called the X-ray adsorption near edge structure (XANES) region. In Fe X-ray absorption spectroscopy, with partially occupied 3*d*-orbitals, XANES is subdivided into the “pre-edge” region (dashed red box in Figure 1A), which corresponds to weak, electric dipole-forbidden but quadrupole-allowed excitations^48,49^ of the 1*s* core electrons into valence 3*d* and charge transfer levels (Figure 1B). These weak transitions can gain intensity via *d*-*p* mixing; see below.^33,34,50,51^ The pre-edge is sensitive to changes in the local coordination environment and oxidation state of Fe.^33,34,43,52^ The main edge (dashed blue box in Figure 1A) arises primarily from the dipole-allowed Fe 1*s* to 4*p* transition (Figure 1B) and reflects the effective nuclear charge (*Z*_eff_) of Fe.^33,34,53^

When the atom absorbs enough energy such that a core electron can be emitted into the continuum (beyond the Rydberg states), the resulting photoelectron undergoes a complex sequence of scattering interactions with the neighboring atoms, resulting in the so-called extended X-ray adsorption fine structure (EXAFS) region (dashed green box in Figure 1A). This region can be used to determine the local geometric structure around the excited atom. A Fourier transformation of this spectral region yields information on the type of and distance to the neighboring atoms.^33,34,51^

In X-ray emission spectroscopy, XES, the core hole that was created in the X-ray absorption process, is filled by electrons from higher energetic orbitals. *K*α (purple spectrum in Figure 1A) and *K*β mainline (i.e., *K*β_1,3_ and *K*β^’^, brown spectrum in Figure 1A) emissions have the same fluorescence initial states with an 1*s* vacancy, but in *K*α and *K*β emissions this vacancy is filled by 2*p* or 3*p* electrons, respectively (Figure 1B). The overall spectral shape of the *K*β emission is dominated by the (3*p*, 3*d*) exchange interaction (Figure 1B), making it extremely sensitive to oxidation and spin state changes, while the 2p spin–orbit splitting shapes the Kα spectra.^33,34,44,45^ Thus, an XANES spectrum reflects the *unoccupied* density of electronic states, while an XES spectrum is related to the density of *occupied* states. By using the X-ray techniques mentioned above, we aim to gain more complete insight into the role of Fe in Ni_*x*_(Fe_1–*x*_)O_*y*_H_*z*_ electrocatalyst under *operando* conditions at different electrochemical potentials. In the text below, we focus on the interpretation and discussion of the Fe site; however, corresponding data for the Ni site are included in the Supporting Information.

## Results and Discussion

### Structural Information from EXAFS and Main-Edge XANES

**Figure 2:**
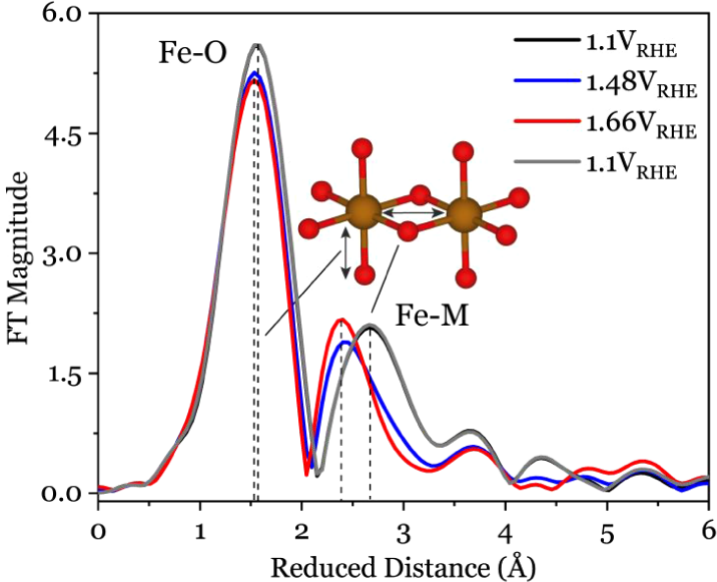
Fourier transformed EXAFS spectra of Fe K-edge for Ni_*x*_(Fe_1–*x*_)O_*y*_H_*z*_ a recorded *operando* at varied electrode biases.

Based on a previous report,^29^ the Ni_*x*_(Fe_1–*x*_)O_*y*_H_*z*_ electrodeposition synthesis protocol employed herein yields layered structures with Ni and Fe centers in edge-sharing octahedral arrangements in accordance with the reported literature.^18,32,54^ These layers have been reported to be separated by intercalating water and ions with a low degree of layer-to-layer long-range ordering which allows for catalytic activity to be distributed throughout the material.^5,9,32,55^ Since the catalytic activity is not limited to the surface, bulk-sensitive techniques such as XAS can readily probe relevant active sites. To identify structural changes in the local environment of the Ni_*x*_(Fe_1–*x*_)O_*y*_H_*z*_ catalyst, *operando* EXAFS measurements were conducted in a 0.1 M KOH electrolyte starting from the resting state (+1.10 V_RHE_) followed by stepping the potential up to the OER catalytic state (+1.66 V_RHE_), and then back down to the resting state (see Figure 1C and *EXAFS simulations using FEFF* section in *SI*). We consider the EXAFS region of the spectrum (signal after Fourier transformation into R*-*space without phase correction), shown in Figure 2, for a quantitative assessment of the bonding arrangement. The Fe EXAFS spectrum at the resting state and potentials up to 1.48 V_RHE_ displays two distinct peaks. Fitting these peaks with FEFF^56^ and SimXLite^29^ reveals octahedral-like coordination with average Fe–O and Fe–M bond lengths of 1.985 ± 0.01 Å 3.050 ± 0.019 Å, respectively. These values align well with those reported in previous studies (Table S1).^18,29,57^ At potentials of +1.48 V_RHE_ and above, the EXAFS peaks shift toward shorter bond lengths without broadening either the Fe (Figure 2) or Ni (see Figure S3) spectral features. This indicates significant bond contraction without a significant change in site homogeneity. Fe–O bond lengths decrease from 1.985 Å (+1.10 V_RHE_) to 1.940 Å (+1.48 V_RHE_) and remain the same (1.938 Å) at the highest applied potential (+1.66 V_RHE_). Notably, these bonds expand to 1.997 Å upon returning to the resting state potential (+1.10 V_RHE_), confirming reversibility (Figure 2).

A strong correlation was observed not only between Fe–O and Ni–O bond lengths (Figures S1–S3) but also between nearest metal–metal distances, which decrease from 3.050 Å (+1.10 V_RHE_) to 2.889 Å (+1.66 V_RHE_). This agrees well with previous *in situ* EXAFS studies reporting contraction in the local metal–O and metal–metal distances.^29,37,57^ Shorter bond distances is usually associated with increased oxidation state or changes in structural symmetry/geometry.^41,42^ However, the absence of a Fe *K*-edge energy shift to higher energies (Figure 4A) has casted doubt on an increase in the Fe oxidation state (from +3 to +4) despite the short Fe–O bond. This contraction may just be a response to the contraction in the NiO_*y*_H_*z*_ host lattice, which will be further explored below.

To quantify the changes observed at different applied potentials, EXAFS fits using the FEFF multiple scattering approaches were performed for both the Fe and Ni *K*-edges, assuming layered Fe-doped structures (see Section S4). The fits employed DFT optimized structures (Figure S5, Table S3 and S4) of NiO_2_, β-, γ-NiOOH, α-, β-Ni(OH)_2_, with 25% Fe doping, which are presented in Figures S7 and S8. As discussed in the Supporting Information, satisfactory fits for the least anodic potential (+1.10 V_RHE_) were achieved with a linear combination fit primarily using α-, β- Ni(OH)_2_ with Fe^3+^Ni^2+^ and β-NiOOH with Fe^3+^Ni^3+^structures (Figures S6–S7 and Table S5). For the most anodic potential (+1.66 V_RHE_), the best fits were obtained with DFT-optimized structures containing 26% of β-NiOOH- Fe^3+^Ni^3+^ (where Fe is in the distorted *O*_h_ symmetry) and 37% of γ-NiOOH-Fe^3+^Ni^4+^ (with Fe in 5-fold coordinated *C*_4*v*_ symmetry). These EXAFS fits support the distorted γ-NiOOH structure at the most oxidizing potentials and a more well-ordered Ni(OH)_2_ structure at resting state potentials. We want to stress, therefore, that all fitted structures have Fe in the 3+ oxidation state. The coexistence of Fe^3+^ in different local geometries is further supported by minor energy difference (2–3 meV per Ni(Fe)O_*y*_H_*z*_ unit from DFT calculations) between γ-NiOOH with Fe^3+^ in 5-fold coordinated *C*_4*v*_ geometry versus *O*_h_ coordination symmetry, indicating that this is a viable interpretation of the atomic structures. Based on these findings, a plausible explanation for the observed EXAFS spectral changes is the oxidation of the Ni scaffold from Ni^2+^ in Ni(OH)_2_ (at +1.10 V_RHE_; resting state) to Ni^3+^/Ni^4+^ in γ-NiOOH with enhanced structural distortion around Fe centers at oxidizing potentials (at 1.66 V_RHE_; catalytic state).

**Figure 3:**
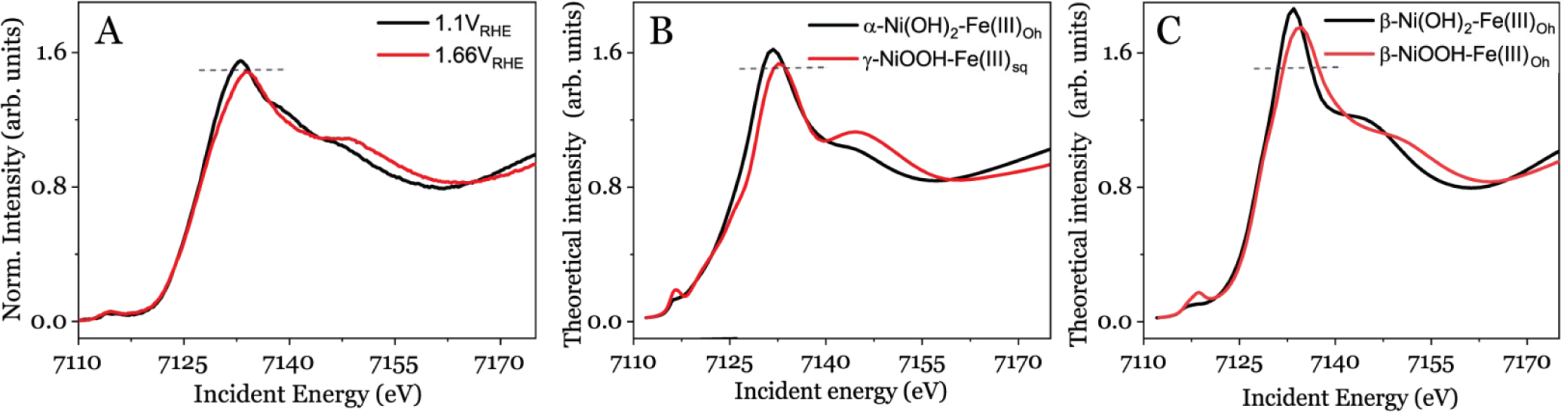
*Operando* experimental (A) and FEFF simulated (B and C) Fe K-edge XAS spectra for different applied potentials The dashed horizontal line guides the eye to the intensity level of the experimentally observed while line at 1.1 V RHE.

Experimental XANES reference data is available only for a five-coordinate *C*_4*v*_ Fe molecular mononuclear complex with N,N,O-tridentate 3,3-bis(1-alkylimidazol-2-yl) propionate ligands, which mimic the active sites of dioxygenase enzymes.^58,59^ However, these ligands differ from the catalytic intermediate proposed in this study. Therefore, we compare our results with simulated spectra, as they better represent the hypothesized structure compared to the reference structures available in the literature. Figure 3 shows measured and simulated XANES spectra for the structures from Figure S5, Tables S3 and S4. The simulated spectra are modeled using the FEFF software that employs a full multiple scattering approach, making it sensitive to the system geometry but less to the electronic structure (e.g., pre-edge features are typically not modeled well),^56^ see Methods. A comparison of the simulated spectrum of α-Ni(OH)_2_ with Fe doping in *O*_h_ symmetry (Figure 3B) matches the intensity of the experimentally observed largest absorption peak (also known as the “white line”) of the Ni_*x*_(Fe_1–*x*_)O_*y*_H_*z*_ in the resting state at 1.10 V_RHE_ (Figure 3A). In contrast, the XANES spectra at +1.66 V_RHE_ in Figure 3A is best matched with the simulated spectra of γ-NiOOH doped with Fe in *C*_4*v*_ symmetry (Figure 3B). This result can, e.g., be realized from the predicted intensity attenuation of the white line and a shift of it to higher energy in the simulated spectra of γ-Ni (Fe)OOH in Figure 3B, which agrees well with the measured XANES spectrum (Figure 3A). This observation aligns well with a modification of the coordination environment of Fe ions occurring at +1.66 V_RHE_. Consistent with the EXAFS data, these XANES results suggest that the observed structural changes involve the shortening of the Fe–O (1.938 Å) and Fe–Ni (2.887 Å) distances. The FEFF-simulated spectra in Figure 3C fit to the experimental observation in Figure 3A but to a lesser degree than Figure 3B due to the broader and more intense “white line” in Figure 3C.

**Figure 4:**
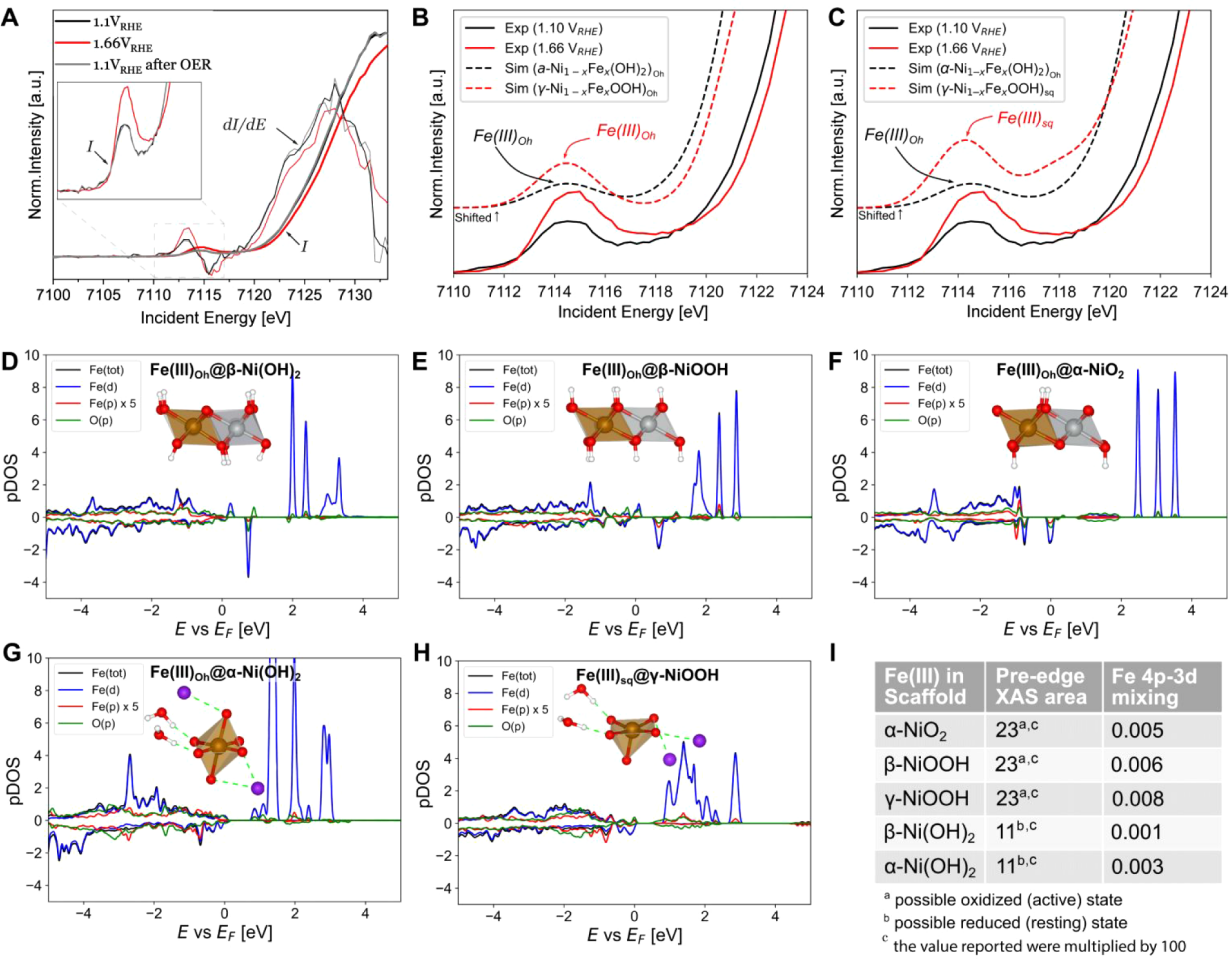
(A) The experimental and first derivative of the normalized XANES data. (B–C) DFT-simulated versus experimental pre-edge XAS. The simulated spectra have been shifted for clarity. (D-H) Core hole density of states (DOS) computed for dilute Fe(III) in different NiO_*y*_H_*z*_ environments. (I) Computed Fe 4p-3d mixing in the XAS valence pre-edge 4d acceptor states versus integrated pre-edge XAS peak area for Fe(III) in different NiO_*y*_H_*z*_ environments. Normalized pre-edge area expressed in 10^–2^ eV units.

In summary, the Fe–O bond length (1.938 Å) at 1.66 V_RHE_ found through the EXAFS analysis is short for Fe(III), even in a distorted *O*_*h*_ geometry. However, the absence of an expected shift to higher energy for both the pre-edge centroid (Figure 4A) and the main edge suggests that the formation of Fe(IV) is unlikely.^60−62^ Instead, the experimental observations (Figures 2 and 3A, and discussion below) could be explained by the loss of one Fe–O bond.^50^ This decrease in coordination number would perturb the symmetry of the site and break the centrosymmetry, leading to geometric transformation from 6-fold coordinated *O*_h_ symmetry to 5-fold coordinated *C*_4*v*_ symmetry. Consequently, the five remaining Fe–O bonds would contract. While EXAFS accurately provides information regarding first-shell Fe–O distances, it determines coordination numbers with the inherent uncertainty of approximately 20% and provides limited information on the site geometry.^63,64^ However, shorter bonds in lower coordination complexes^65−68^ also contribute to increased 4*p* mixing.^50,69^ Therefore, in the next section, we explore in more detail how the electronic structure of various Ni_*x*_(Fe_1–*x*_)O_*y*_H_*z*_ states corresponds to changes in the pre-edge XANES spectra, which are sensitive to both the oxidation state and the geometry of the Fe atoms.^69−71^

### Analysis of the Adsorption Pre-Edge by HERFD XAS

Probing the XAS pre-edge region of transition-metal centers has been demonstrated as a versatile tool for examining the electronic structure of electrocatalysts *in situ*.^35,72^ In general, the pre-edge energy position is determined by the energy of the metal *d*-band states and the core 1*s* orbital, while the main edge position relates the energy between the Fe 1*s* and 4*p* orbitals. A linear relationship has been found between both the edge and pre-edge positions with atomic charge as well as effective nuclear charge (*Z*_eff_).^51,60^ Therefore, the oxidation state change of the absorbing metal center, which is closely tied to *Z*_eff_, directly affects the energy positions of the edge and the pre-edge. For instance, since *Z*_eff_ of the absorbing atom typically increases if oxidation occurs, both the edge and the pre-edge will shift to higher energy. This is because the 1*s* core level states experience a stronger influence from changes in *Z*_eff_ relative to the more weakly bound and more screened valence states. As *Z*_eff_ increases, the energy gap between the core and the valence level increases resulting in shifts of the pre-edge and the rising edge to higher energies. Contracted bond distances observed in the EXAFS (Figure 2) are commonly associated with an increased oxidation state. Such oxidation state increases might also be seen in the Fe XANES spectra, where the fine structure of the peaks contains valuable information about the electronic states of the sample.

Fe *K*-edge (1*s* to *3d* or 4*p* transitions) XANES has been extensively employed to probe electronic structural changes in OER electrocatalysts.^72^ However, with traditional XANES methods, the short 1*s* core hole lifetime^73,74^ leads to spectral broadening and a loss of chemical information. This limitation can be overcome by using high-energy resolution fluorescence detected (HERFD) XANES.^45,74,75^ By selectively measuring the *K*α_1_ fluorescence line (2*p*_3/2_ →1*s*) using high-resolution Bragg optics,^45,76−78^ the effective broadening of the 1*s* core hole lifetime can be suppressed. Employing this technique, the chemical information lost in the TFY (Total Fluorescence Yield) spectrum can be recovered, as demonstrated in the HERFD Fe spectrum in Figure S9.

Fe HERFD recorded at potentials ranging from +1.10 V_RHE_ to +1.66 V_RHE_ are presented in Figure 4A. The pre-edge features do not change their energy positions, which is strikingly different from previous experimental observations for Fe complexes in various oxidation states and, notably different from oxidation of the Fe from Fe(II) to Fe(III) or Fe(IV).^60,62,79−81^ As for the pre-edge, the energy position of the main edge remains constant between +1.10 V_RHE_ and +1.66 V_RHE_ potentials (inflection points in the first derivative of the normalized HERFD data Figure 4A), as also seen in the literature.^18^ Accordingly, *Z*_eff_ is constant in contrast to what is expected upon oxidation of the Fe centers. This observation corroborates our interpretation that spectral changes seen for different potentials are associated with changes in the ligand field around Fe centers, resulting in ligand field splitting caused by local geometric variations. If an oxidation state change occurred, the change in Z_eff_ would have needed to affect the energy of the 1*s* and 4*p* states to a similar degree in order to match the obtained HERFD spectra, which is possible but highly unlikely.

The 1*s*-3*d* pre-edge feature is also sensitive to the ligand geometry around the Fe center. Generally, a larger pre-edge intensity with no change in energy positions is associated either with (i) symmetry distortion due to deviation from the centrosymmetric coordination environment leading to asymmetric bond lengths or (ii) a decrease in coordination number from the centrosymmetric *O*_h_ with a decrease in the coordination number. In the latter case, the rule of thumb postulates that the *O*_h_ (6-coordinated) environment corresponds to the weakest pre-edge intensity with increasing intensity for lower coordination numbers (see inset Figure 4A–C).^44,50,60,69,71^ As the symmetry becomes more distorted, the pre-edge peak area increases. This increase in intensity is assigned to mixing metal 4*p* into the 3*d* orbitals, which adds some dipole-allowed 4*p* character to the usually dipole forbidden (but quadrupole allowed) pre-edge transition (1*s*-3*d+*4*p* transition with *d-p* mixing). This yields an increased intensity even at small admixtures, since electric quadrupole-allowed transitions are about 100-times weaker than electric dipole-allowed transitions.^48^ As a result, only a few percent of 4*p* mixing into the 3*d* orbitals can significantly impact the intensity of the 1*s*-3*d* pre-edge feature. This correlation has been utilized in numerous studies, not only for Fe,^50,69,71^ but also for other transition metals, such as Mn, Co, Ni, and Cu, in relation to their geometry and coordination number.^53,82−84^

In a centrosymmetric six-coordinated environment, 1*s*-3*d* pre-edge intensity can only be due to the weak electric quadrupole mechanism since mixing of the *gerade* 3*d* orbitals with the *ungerade* 4*p* orbitals is not allowed. From previous work, the pre-edge intensity of 23 *O*_h_ Fe(III) complexes,^50,69^ is on average 6.2 eV units (note that here and below normalized pre-edge area expressed in 10^–2^ eV units) with the postedge continuum normalized to unity. The pre-edge here consists of two features split by the crystal field parameter 10Dq. Since in high spin Fe(III), the five d-electrons each singly occupy the orbitals, the ground state spectroscopic term ^5^A_1g_ has a (t_2g_)^3^(e_g_)^2^ character that produces two excited hole configurations (t_2g_)^2^(e_g_)^2^ and (t_2g_)^3^(e_g_)^1^ upon promotion of 1s into 3d unoccupied states. This results in final states of ^5^T_2_ and ^5^E characters (i.e., two peaks split by 10Dq), respectively, as observed in previous studies.^50,85−87^ In contrast, the pre-edge area observed for the resting state of the Ni_*x*_(Fe_1–*x*_)O_*y*_H_*z*_ catalysts at a potential of 1.10 V_RHE_ is equal to 11 units and does not have a pronounced multiplicity of the peak. This experimental observation of increased area can be adequately explained from 4*p* mixing into 3*d* orbitals, leading to a gain of intensity through additional electric dipole transitions. Assuming that the pre-edge area of 6.2 units (as determined from the *O*_h_ Fe(III) complexes, see above) represents the total quadrupole intensity, the remaining pre-edge area in the Ni_*x*_(Fe_1–*x*_)O_*y*_H_*z*_ at a potential 1.1 V_RHE_ should be attributed to the amount of electric dipole intensity. Therefore, the dipole intensity of the pre-edge feature can be estimated to be 4.8 units (11–6.2), which originates from the 4p character in the 3d orbitals. The relationship between the intensity of dipole contribution to the pre-edge intensity and the amount of 4*p* character in the final state for Fe(III) is well established and corresponds to 1.5 units for a 1% increase in Fe(III) 4*p* character.^50,53^ Therefore, the contribution of Fe 4*p* character to the unoccupied valence 3d orbitals is estimated to be 4.8/1.5 = 3.2% for Ni_*x*_(Fe_1–*x*_)O_*y*_H_*z*_ at potential 1.10 V_RHE_.

Both dimetal Fe(III) oxides^44^ and six-coordinate μ-oxo-bridged dimetallic Fe(III) complexes^50,69^ also exhibit similar single pre-edge structure and a similarly sized area within the range of resting state of Ni_*x*_(Fe_1–*x*_)O_*y*_H_*z*_ at a potential of 1.10 V_RHE_. The interpretation there is that the Fe site has distorted *O*_h_ symmetry and the distribution is understood by invoking 3*d*-4*p* mixing into the orbital component along the distortion. The inversion symmetry is broken and allows mixing between the 3*d* and the 4*p* orbitals (both axial distortions along the *z*-axis and the equatorial plane permit mixing of the same symmetry 3*d* and 4*p* orbitals and are allowed to mix by the group theory).^50,85−87^

The pre-edge gains intensity by a factor of ∼2 upon applying OER potentials (areas at +1.10 V_RHE_ = 11 and +1.66 V_RHE_ = 23). This indicates a significantly more covalent bonding at +1.66 V_RHE_ and even more increased 3*d*-4*p* mixing than in the resting state potential of +1.10 V_RHE_. Based on the assumption stated earlier, the percentage of Fe 4*p* mixing into the Fe 3*d* orbitals is thus estimated to be (23–6.2)/1.5 = 11.2% for OER active Ni_*x*_(Fe_1–*x*_)O_*y*_H_*z*_. A plausible explanation for the gain in pre-edge intensity at +1.66 V_RHE_ compared to +1.10 V_RHE_ without a shift to higher energy is a geometric transformation from distorted six-coordinated sites to a five-coordinated environment or to a mixed state with both five- and six-coordinated Fe. Such explanations also find support in the literature^50,69^

The loss of a ligand increases the amount of total 4*p* mixing in Fe(III) complexes (experimentally determined 4*p* character at +1.10 V_RHE_ = 3.2% and +1.66 V_RHE_ = 11.2%) and is attributable to the generally shorter bond lengths, thereby accentuating geometric perturbations. A shorter Fe–O bond correlates with the decrease in coordination number, which is associated with a larger mixing of Fe 4*p* states into the Fe 3*d* orbitals, and gives rise to an electric dipole that allows Fe 1*s*-3*d+*4*p* transition at +1.66 V_RHE_. This correlation is also supported by the EXAFS data in Figure 2.

To further investigate the origin of the spectral features of the pre-edge, DFT-simulated XAS spectra were computed for a library of structures containing Fe in various environments (Figure 4B,C). In this way, plausible structural environments can be tested with the best fits kept for further evaluation. The encompassed structures are based on α-,β-Ni(OH)_2_, β-,γ-NiOOH, and α-NiO_2_ with doped Fe forged into symmetric and asymmetric *O*_h_, *C*_4*v*_, and *T*_d_ (shown in Figures S10–S12). Ligand environments within the NiO_*y*_H_*z*_ scaffold structures were further varied by either adjusting O/OH group ratio (thus allowing for variation of the local oxidation state from II to IV), or by substituting one O/OH group by water, epoxide, or peroxide groups. The full data set is presented in the *SI*. At the resting state of the catalysts, the best fits versus experimental pre-edge data is found for a high-spin Fe(III) oxidation state in a local FeO_3_(OH)_3_ O*_h_* coordination environment within an α-Ni(OH)_2_ scaffold (Figure 4B,C). An Fe(III) center in a distorted *O*_h_ FeO_3_(OH)_3_ coordination, but residing within a γ-NiOOH scaffold, also provided a reasonable match for the data representing the catalytically active state (Figure 4B). Nevertheless, for the data acquired at +1.66 V_RHE_, a closer match between experiment and simulations was found for a *C*_4*v*_ FeO_4_(OH)_1_ coordination around a Fe (III) center (Figure 4C).

Following the procedure of Juhlin et al.,^36^ who studied pre-edge features of Co in LiCoO_2_, we can further investigate the origin of the pre-edge for Fe(III) by decomposing the peak into dipolar and quadrupolar components. Although both components give non-negligible contributions, the dipole contributions explain the majority of the peak (as noted above). The dipolar transition becomes allowed due to the significant mixing of the 3*d* Fe with the Fe 4*p* states and, to a lesser extent, the O 2*p* states (see Figure 4D*–*H). For the oxidized Ni_*x*_(Fe_1–*x*_)O_*y*_H_*z*_ structures at OER conditions, the *p*-*d* mixing effect is enhanced by the asymmetry introduced by the 5-fold coordination environment with mixed O and OH ligands. In addition, the effect becomes more apparent as the NiO_*y*_H_*z*_ scaffold surrounding the Fe(III) site becomes further oxidized, leading to a Fe–O bond contraction. In other words, in line with the experimental results discussed above and ligand field theory, the covalency increases as the catalyst shifts from its resting to active state. Consequently, the amount of Fe 4*p* and O 2*p* mixing in the Fe 3*d*-dominated band gap state close to the Fermi-level scales with the area under the pre-edge peak (see Figure 4I). The larger *p*-mixing (cf., Figure 4H) observed in the active states results in a larger pre-edge area (Figure 4I).

### Spin State and Effective Core Potential Probed by the XES K-Edge Spectra

To further validate our interpretation of the HERFD XAS data, we employ XES spectroscopy to probe both the Kα and Kβ emissions. Together, the XES spectra serve as a robust test for the consistency of our hypothesis, which suggests a nonoxidative local structural change in the Fe geometry. The *K*α XES probes the 2*p* to 1*s* transition and is split into *K*α_1_ and *K*α_2_ peaks due to spin–orbit interactions. It was reported that the full width at half-maximum (fwhm) of the *K*α_1_ line is roughly proportional to the “nominal” number of unpaired 3*d* electrons,^88−91^ i.e., the number of unpaired 3*d* electrons as derived from the formal oxidation state of the metal atom. This proportionality is usually explained within the framework of multiplet theory by assuming that the *K*α_1_ line is broadened by the exchange interaction between the 2*p* hole in the final state and the populated 3*d* orbitals.^33,74,92^ Therefore, it reflects the net spin located at the metal atom. However, the 2*p*-3*d* exchange interaction is weaker than the spin–orbit interaction of the 2*p* level and the spin-polarized nature in *K*α is manifested as a slight asymmetry hidden within the single *K*α_1_ and *K*α_2_ peaks.^44,45^ Nonetheless, a roughly linear relation between the fwhm of the more intense *K*α_1_ peak and the nominal spin value given by the 3*d* electrons has been established.^44,45,88−91^

**Figure 5:**
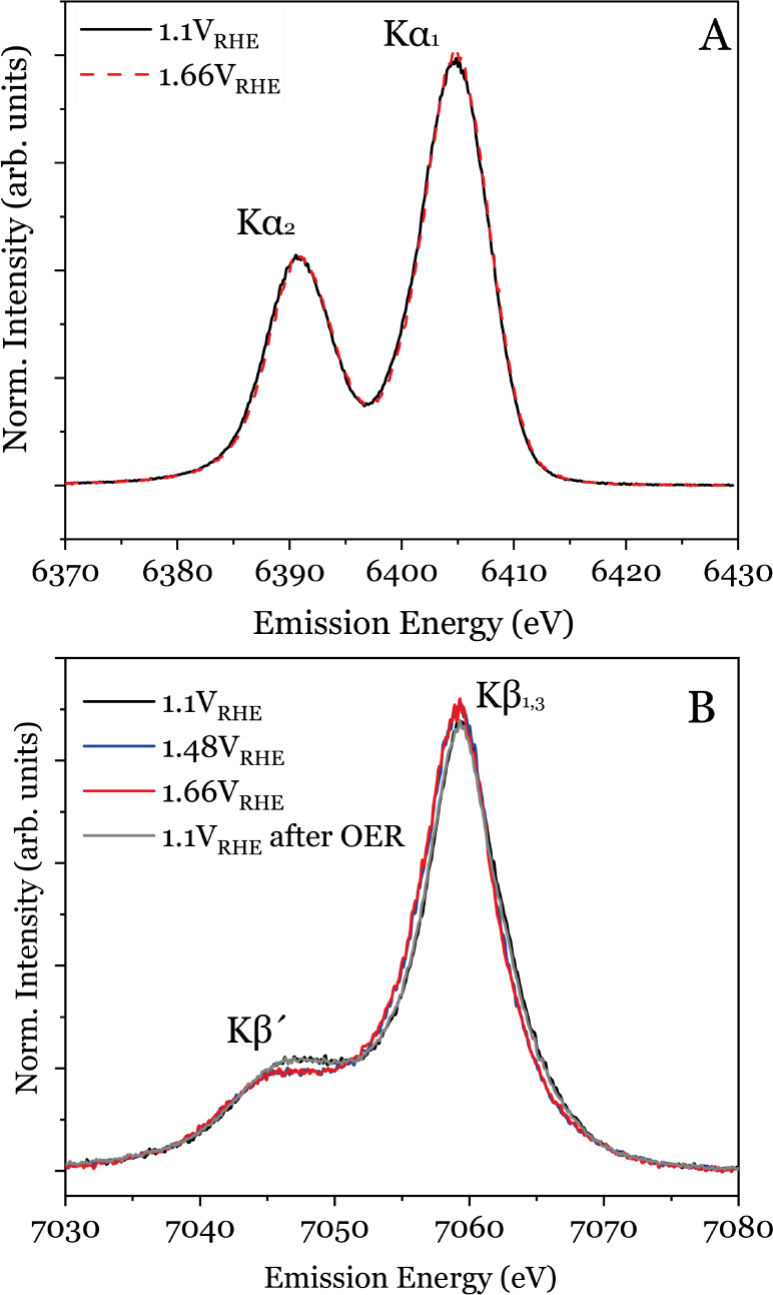
(A) Fe Kα XES spectra of Ni_*x*_(Fe_1–*x*_)O_*y*_H_*z*_ at 1.10 and 1.66 V_RHE_. (B) Fe Kβ main line XES spectra of Ni_*x*_(Fe_1–*x*_)O_*y*_H_*z*_ cycled from 1.10 V_RHE_ to 1.66 V_RHE_ and back to 1.10 V_RHE_ after OER. See Supporting Information for the full data set.

It is known that Fe-sites remain as high spin Fe(III) (S = 5/2) below the oxidation threshold potential of Ni(OH)_2_/NiOOH at ∼1.35 V_RHE_.^30,41^ High-spin Fe(II) and Fe(IV) both have a S = 2 spin state. Thus, if the Fe transitions to a different spin or oxidation state occur leading to a change in the number of unpaired 3*d* electrons, it would be reflected in the fwhm of the *K*α XES spectra. As seen in Figure 5A comparing the Fe *K*α XES spectra at 1.10 and 1.66 V_RHE_, no spectral change associated with a change in oxidation or spin state is noted. This again suggests that the spectral changes seen in the XANES and EXAFS spectra (Figures 2, 3A and 4A) are not related to oxidation nor spin state changes and that Fe remains in the same state within the considered potential range. Instead, *K*α spectra is in line with previously reported effects of geometry changes for high-spin Fe(III) complexes.^44^

**Figure 6:**
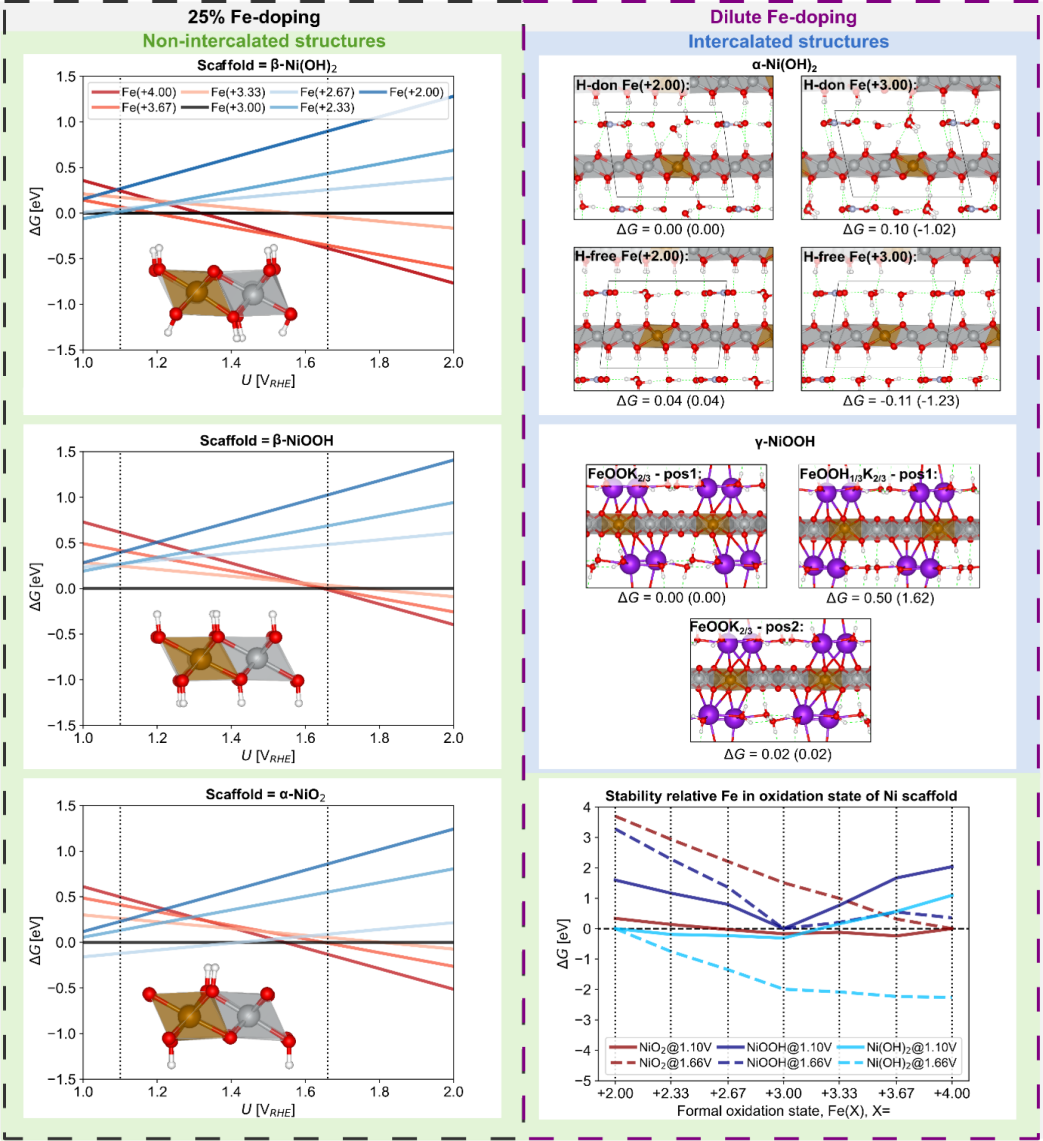
DFT optimized structures and relative energies. To the left, bulk free energies per metal unit (Δ*G*) of 25% Fe-doped NiO_*y*_H_*z*_ nonintercalated scaffold structures as a function of potential for varied Fe oxidation states. Inserts show a single layer of optimized Fe(III) structures. To the right, dilute Fe-doping. Top right, intercalating α-Ni(OH)_2_ with Fe doped at two different positions: “H-don” (H-donating) where all the OH groups are donating H-bonds to the intercalating water, and “H-free” where two OH are not participating in the H-bonding network. These energies correspond to Fe(II) are compared to Fe(III) structures with corresponding energies in brackets. Middle right, γ-NiOOH with Fe doping at two positions. Although this state is formally Fe(+3.67), its magnetic moment (4.25) and Bader charge (1.71) best corresponds to a Fe(+3.00) state.^94^ These are compared to a structure with two added H, yielding a formal Fe(+3.00) state. Lower right, nonintercalating low Fe-doping (6%): relative free energy at resting and activated state potentials versus formal oxidation state.

The relative peak shape and position of the *K*β fluorescence transition (3*p* to 1*s*) is dominated by the 3*p*–3*d* exchange interaction that is stronger than the 2*p*-3*d* interactions of *K*α XES. This makes *K*β XES much more sensitive to local spin magnetic moments in the 3*d* shell as well as changes in the number of unpaired 3*d* electrons than the *K*α XES.^44,45^ The *K*β spectrum comprises a main intense peak, *K*β_1,3_, and a low energy shoulder, *K*β_’_. Whereas the main peak *K*β_1,3_ mainly reflects transitions from 3*p* that have spin-states of opposite direction to the majority of the 3*d* spin states, the satellite *K*β_’_ arises from transitions from states of the same spin direction.

It is known that the main *K*β lines (as discussed in the Introduction) are much more sensitive to the metal spin and geometry state change than the *K*α lines. In Figure 5B, we compare the spectra of Ni_*x*_(Fe_1–*x*_)O_*y*_H_*z*_ as a function of the applied potential. All spectra show Fe in high-spin states, which is concluded from the presence of the *K*β’ feature that would otherwise shift to higher energies and merge with the main feature around the *K*β_1,3_ peak for low-spin states due to the weak 3*d* spin moment. At the potential of 1.48 V_RHE_Figure 2 the *K*β spectra show a slightly less pronounced *K*β’ shoulder and the main *K*_β1,3_ peak shift toward lower energy with respect to the potentials below +1.48 V_RHE_ (see Supporting Information for the full data set). The spectral signatures corresponding to the bond length contractions in the EXAFS data occur at the same potential here (Figure 2). Again, as in all the above experiments, the changes in the main *K*β lines are fully reversible.

It is a reasonable hypothesis to attribute the observed spectral changes to spin and/or oxidation state changes, as previously reported for Fe and other 3*d* transition metals^44,45,88,93−95^ To evaluate the *K*β spectral evolution, we have performed an IAD (Integrated absolute difference, see Figure S14) analysis.^88^ As suggested by Glatzel and coworkers^44^ this is carried out after aligning the spectra to have the same center of gravity (COG) to eliminate the energy shift due to the screening of the core-hole potential, which depends on the valence charge density. We assume the same high-spin 3*p*^5^3*d*^5^ final state for all applied potentials. The resulting IAD analysis yields identical results with or without the COG alignment (Figure S14). This implies that the shift of the *K*_β1,3_ peak is not caused by variations in the electronic screening that arise from changes in the valence charge density between different oxidation or spin states but can be attributed almost entirely to the 3*p*-3*d* mixing.^44^

The following discussion provides further support for our interpretation of the constant formal Fe(III) oxidation state with changes occurring only through symmetry breaking and bond contraction around the Fe ion. As the splitting between the *K*β mainline features is governed largely by the 3*p*–3*d* exchange integrals, it has, e.g., been found that the split is modulated by the metal–ligand covalency for, e.g., Fe, Co, and Mn in both *O*_*h*_ and *T*_*d*_ coordination.^44,46,96^ Experimental studies have reported a reduced *K*β′–*K*β_1,3_ splitting for Fe with an increase in the covalent character in the *K*β spectra of the compounds having the same nominal spin^46,94^ The herein observed 0.1 eV shift in the first moment of the *K*β_1,3_ peak between +1.10 V_RHE_ and +1.48 V_RHE_ (as shown Figure 5B, with details provided in the SI) is in agreement with the shifts found for covalency increase.^44,46^ Therefore, a plausible explanation is that geometry changes in Ni_*x*_(Fe_1–*x*_)O_*y*_H_*z*_ toward noncentrosymmetric structures for more oxidizing potentials above 1.48 V_RHE_ causes an enhanced *p*-*d* mixing between ligand and metal.

The absence of inversion symmetry in square pyramidal coordination enhances orbital mixing. From the character table for the *C*_4*v*_ point group, we note that Fe *p* and some *d* orbitals have A_1_ and E symmetry, as do some orbitals formed by O/OH ligand *p* orbitals. This means a stronger *p*-*d* mixing between ligand metal orbitals at a potential of 1.48 V_RHE_ compared to distorted *O*h symmetry in the resting state (see Analysis of the adsorption pre-edge by HERFD for more detailed information). Thus, a change in the local surrounding environment (coordination number; geometric transformations from *O*_h_ to *C*_4*v*_) of Fe during OER leads to spectral modification through the altered degree of covalency of the metal–ligand (Fe–O) bond. This spectral response to coordination environment changes is observed not only in EXAFS as well as in experimental and theoretical HERFD but also in the *K*β spectra (Figure 5B) in favor of the interpretation of *C*_4v_ symmetry around Fe(III) in the active OER catalyst. Another argument suggesting that the *C*_4*v*_ coordination has a more covalent character than that of *O*_h_ is the shorter bond distance in the former^44^ (see, also, EXAFS data above). In the ligand field depiction of *C*_4*v*_ compared to *O*_h_ coordination, the high spin Fe(III) center has more unpaired electrons in the *d* orbitals of e and a_1_ symmetries. Orbitals of these symmetries tend to form ligand bonds with more covalent character than the other orbitals that transform to b_2_ and b_1_ symmetries, as found in *O*_h_ coordination. Similar *K*β spectra evolution for different local coordination environments was reported by Lafueza et al.^44^ for Fe(III) and by Peng et al.^74^ for Mn(II), which is an isoelectronic (3*d*^5^ configuration) to Fe(III) compounds. The situation is reversed for the *O*_h_ coordination case (see Analysis of the adsorption pre-edge by HERFD XAS for more details).

The conclusions drawn from the Kβ analysis align with the Fe Kα results. Specifically, the absence of significant changes in the Kα spectra between the active and resting states of the electrocatalyst material (Figure 5A) is consistent with previously reported effects of geometric changes in high-spin Fe(III) complexes.^44^ These findings suggest that the observed spectral differences are attributable to structural rearrangements rather than changes in spin or oxidation state, as further elaborated in the Supporting Information.

### Energetics from DFT Calculations

In order to shed further light on the most likely state of Fe under resting and operational conditions, DFT calculations were carried out comparing the energetics of different Fe-doped model structures. Models evaluating energetic trends include dilute (∼6%) Fe in nonintercalating α-NiO_2_, β-NiOOH, and β-Ni(OH)_2_ as well as fully H_2_O and ion (K^+^ or NO_3_^–^) intercalated α-Ni(OH)_2_ and γ-NiOOH type structures with 10% Fe.^32,97^ We also studied increased Fe-doping (25%) for the nonintercalating structures. The higher concentrations of Fe better represent the average expected Fe concentration from the experimental synthesis protocol. However, such higher concentrations do not allow for full control over the effects on the Fe center from varying the NiO_*y*_H_*z*_ scaffolding environment as any change in the Fe structure or oxidation simultaneously affects the NiO_*y*_H_*z*_ scaffold. Thus, we use the low Fe-concentration models to gain valuable insights into the influence of the scaffold under a constant Fe oxidation state (as modeled by the number of O/OH ligands). In addition, although fully intercalated structures are expected from the experiments, there are large degrees of freedom in these structures. The ion-impregnated intercalating aqueous layer can be arranged in multiple ways. This warrants a complementary systematic study of more well-defined nonintercalating structures as a comparison to the calculations with intercalating models. We also evaluate the formation of Ni_*x*_(Fe_1–*x*_)O_*y*_H_*z*_ with Fe in a *C*_4*v*_ compared to *O*_h_ coordination environment for the active catalyst under OER potentials.

The high-spin state was always found to be the most favorable for Fe in Ni_*x*_(Fe_1–*x*_)O_*y*_H_*z*_.^30,31,98^ For Ni, the Ni(+2.00) structure is in a triplet states, whereas Ni(+3.00) and Ni(+4.00) are low spins.^20,41^ (Note that we use numerical values in this section to allow for noninteger, average, oxidation states.) In the general case, both Fe and Ni prefer a ferromagnetic (FM) internal spin arrangement in the Ni_*x*_(Fe_1–*x*_)O_*y*_H_*z*_. structures (note that this is not always the case for pure Ni samples) with the Fe and Ni in a mutual antiferromagnetic (AFM) arrangement. This ordering was also found for the NiO_2_ scaffolds, although the Ni magnetic moments are close to zero. For the nonintercalating structures and for α-Ni(OH)_2_, local magnetic moments and computed Bader charges for the compounds followed the expected values corresponding to a given Ni or Fe formal oxidation state as reported by Zhao et al.^99^ γ-NiOOH was the exception where Fe in the formal Fe(+3.33) and Fe(+3.67) positions both give magnetic moments (4.25). Bader charges (+1.71) that best corresponds to Fe(+3.00). This indicates that Ni prefers to carry the excess charge beyond +3.00 so that Ni is oxidized while Fe remains Fe(+3.00).

Figure 6 compares the energetics at varied potential biases for different Ni_*x*_(Fe_1–*x*_)O_*y*_H_*z*_ structures with Fe in *O*_h_ symmetry. Starting from the 25% Fe-doped nonintercalating model structures, Figure 6 (left) shows the relative free energy per metal atom (Δ*G* = Δ*G*/*n*, with *n* being the number of metal atoms in the model) of Fe at +2.00 to +4.00 oxidation state in different nonintercalated NiO_*y*_H_*z*_ scaffolds as a function of potential. Energies are given relative to Fe(+3.00). At *U* = 1.10 V_RHE_, Fe(+3.00) is favored for all scaffold structures except in the NiO_2_ in which Fe(+2.67) is preferred. As the potential is increased, more oxidized states of Fe become favored. For the Ni(OH)_2_ scaffold, this happens early at 1.2 V_RHE_, for NiOOH at 1.65 V_RHE_, and for the NiO_2_ scaffold at 1.5 V_RHE_. Hence, at low potential corresponding to the catalyst resting state, a Fe(+3.00) state is predicted, whereas the transition from Fe(3.00) to Fe(4.00) could occur before or just after the 1.66 V_RHE._ We note that the transition could be kinetically limited and that an applied overpotential exceeding the predicted reversible potentials might be needed to spark the oxidation, explaining the lack of the inferred oxidation state experimentally. Figure S15 compares the energetics of all oxidation states in all nonintercalated NiO_*y*_H_*z*_ scaffolds. This indicates that Ni(OH)_2_ and NiO_2_ are favored at the 1.10 V_RHE_ and 1.66 V_RHE_ potentials, respectively, with Fe likely in a Fe(+3.00) state.

Comparing the above results to the low Fe-doping case gives a similar picture with few exceptions. As above, in the Ni(OH)_2_ scaffold and at 1.10 V_RHE_, Fe prefers a Fe(+3.00) oxidation state, but at the higher potential, it becomes Fe(+4.00). In the NiO_2_ scaffold, an oxidation state between Fe(+3.67) to Fe(+3.00) is favored, but Fe(+4.00) is preferred at the 1.66 V_RHE_ potential. In contrast to the above, for the NiOOH scaffold, the Fe(+3.00) is preferred throughout, which is best explained by the large penalty of breaking the H-bonding network to create Ni(Fe)O_2_.

In addition, we need to account for the effect of intercalation between the Ni_*x*_(Fe_1–*x*_)O_*y*_H_*z*_ layers. As stated before, intercalated structures are expected experimentally, and the lack of direct interactions between the Ni_*x*_(Fe_1–*x*_)O_*y*_H_*z*_ layers could influence the relative energetics of different oxidation states significantly. For α-Ni(OH)_2_, we create a NO_3_^–^ intercalated structure based on the experimental conditions and previously optimized structures of Dionigi et al.^32^ Fe doping was accounted for with Fe evaluated in all unique Ni positions. While the energy difference for Fe(+2.00) was moderate between the different positions (±0.1 eV), the most favorable positions for creating Fe (+3.00) are shown in Figure 6; creating Fe(+3.00) is favorable relative to other oxidation states (and positions) if Fe is placed in an initial environment where two OH ligands lack H-bonds to intercalating water. Shifting these OH into O (plus another previously H-bonded OH, yielding three O in total) to create Fe(+3.00) is favorable at low potentials (1.10 V_RHE_), but not at higher potentials, which is in line with our results from the nonintercalating models. For γ-NiOOH, the intercalating layer contains K^+^ and water.^29,97^ As no H are part of the structure, the amount of K^+^ sets the formal oxidation state to Fe (+3.67) and Ni(+3.67) on average. From these structures, as shown in Figure 6 for the lowest energy cases, adding additional H around Fe to create a formal Fe(+3.00), is not beneficial. This is likely influenced by the disrupted H-bonding network upon addition of extra H. Nevertheless, an alternative interpretation is that Fe is already Fe(+3.00) in the γ-NiOOH state, and that Ni centers carry the excess charge left from Fe; hence Ni is approximately Ni(+4.00) while Fe is Fe(+3.00). This interpretation is also supported by the computed local magnetic moment and Bader charge of the Fe (see above) that favors a Fe(+3.00) oxidation state based on a comparison to previous reports.^99^

For the case of *O*_h_ coordination environments evaluated above we can draw a few conclusions; whereas the DFT energetics results do not fully resolve the character of Fe in Fe-doped NiO_*y*_H_*z*_, they do favor a picture where Fe(+3.00) oxidation state is preferred at the lower applied potentials (∼1.10 V_RHE_) and that the stability region of Fe(+3.00) likely stretches up to 1.66 V_RHE_. The latter finding is also feasible for the γ-NiOOH fully intercalated structure that, based on previous experimental findings, is expected to be the prevailing structure at 1.66 V_RHE_.^20^ Therefore, together with the reasonable agreement between experimental and simulated XANES pre-edge and EXAFS data, a plausible interpretation is that the character of Fe is predominantly Fe(+3.00) in an *O*_h_ environment at OER operation conditions. In this picture, the spectral changes in XAS and XES data compared to the resting state can be explained by a variation in the local coordination environment that is a direct consequence of varying O/OH bonds in the NiO_*y*_H_*z*_ scaffold. While the above conclusion cannot be rejected, the combined support for Fe(+3.00) in a square pyramidal 5-fold coordination (*C*_4*v*_) as the active state in OER catalysis is deemed stronger, as further explained below.

**Figure 7:**
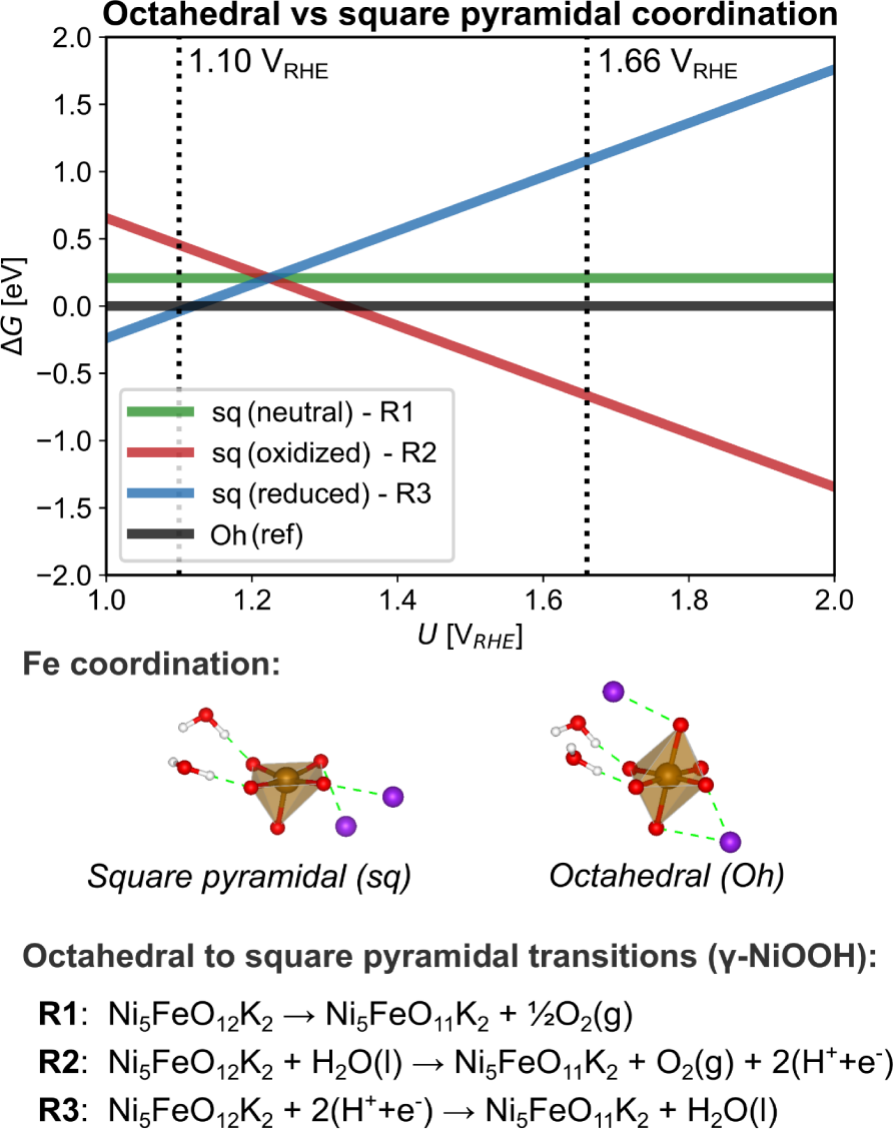
DFT results for Fe-doped γ-NiOOH with Fe as Fe(III) in *C*_4v_ coordination. Energetics comparing *O*_h_ to *C*_4v_ coordination around Fe through reaction R1–3 at various resting to OER active potentials.

Lastly, we address the possibility of generating an active Fe state in a *C*_4*v*_ symmetry and compare it to the picture above in which Fe remains Fe(+3.00) in an *O*_h_ coordination environment. Figure 7 shows the free energy of forming a local FeO_5_ structure from FeO_6_ in γ-NiOOH by either a neutral/chemical (R1), oxidative (R2), or reductive (R3) reaction. In our model, Fe-doped γ-NiOOH is simulated by the water and K^+^ intercalated Ni_5_FeO_12_K_2_ structure (same as in Figure 6.) that evolves into Ni_5_FeO_11_K_2_ through the simultaneous formation of O_2_ or H_2_O (see R1–3 in Figure 7). We find that the distorted *O*_h_ reference structure is preferred at potentials below ∼1.25 V_RHE_, whereas oxidative formation of the *C*_4*v*_ is preferred at higher potentials corresponding to the potentials probed during OER evolution. Square pyramidal intermediate states are indeed part of the majority of the proposed catalytic mechanisms of *O*_2_(*g*) formation using Ni_*x*_(Fe_1–*x*_)O_*y*_H_*z*_ electrocatalysts.^100,101^ As O_2_ evolution is ongoing at these potentials, it is thus reasonable to assume the *C*_4*v*_ structure in Figure 7 is kinetically accessible. Figure 4C also demonstrates that the simulated XANES pre-edge spectra is compatible with the experimental results; the pre-edge consists of a single component and its intensity is significantly increased compared to the resting. In addition, the EXAFS spectra simulated for the *C*_4*v*_ state are also in good agreement with experimental data (Figure 4A). Hence, we conclude that Fe(+3.00) in a *C*_4_*_v_* coordination–possibly in dynamic equilibrium with *O*_h_ Fe(+3.00) – is a likely active state for the Ni_*x*_(Fe_1–*x*_)O_*y*_H_*z*_ catalysts material at OER potentials.

## Conclusion

In this work, we have employed a host of X-ray spectroscopy techniques, including HERFD XANES, EXAFS, as well as *K*α and *K*β XES, to examine the electronic and local structural evolution of the Fe site in Ni_*x*_(Fe_1–*x*_)O_*y*_H_*z*_ electrocatalyst *in situ* at different electrochemical potentials. These range from a resting state (1.10 V_RHE_) to oxidized and catalytically active (1.66 V_RHE_) states of the electrocatalyst. Our results give a detailed picture of the equilibrium state of the catalysts under the applied conditions. At the resting state, the Fe-doped NO_3_^–^(aq) intercalated α-Ni(OH)_2_ phase matched best the combined experimental and DFT results, while under catalytic conditions, the best match is represented by Fe-doped γ-NiOOH with intercalated and solvated K^+^(aq) ions. Moreover, through experimental observation and comparison to DFT electronic structure simulations, we are able to assign variations in spectral features to different Fe local coordination environments that correspond to changes in the degree of *p*-*d* mixing between O/OH ligand 2*p* states and the 3*d* and 4*p* states of Fe. More specifically, we find that the chemical picture of the Ni_*x*_(Fe_1–*x*_)O_*y*_H_*z*_ catalysts that best fits the observed results is a FeO_*y*_H_*z*_ center that exhibits substantial distortions from the *O*_h_ geometry (found at the resting state potential) with Fe remaining at all potentials at a formal Fe(III) oxidation state within the studied 1.10–1.66 V_RHE_ potential range. Spectral changes are associated with alterations in the covalency of the Fe–O bonds. These changes are induced by O ligand dissociation leading to a local geometry change from distorted *O*_h_ to *C*_4v_ and a simultaneous oxidation of the NiO_*y*_H_*z*_ scaffold caused by the applied potential bias. This surprising result allow us to speculate that the Fe active site under OER operation has a weakly coordinated or easily dissociable ligand. The active site would then have the flexibility to be five- or six-coordinate, depending on the applied potential, consistent with its catalytic function. An easily dissociable ligand would provide the required substrate oxygen for O–O bond formation and readily available sites during the catalytic cycle to bind intermediate species.

## Supporting Information Available

Additional figures and a table that provide further details on sample preparation, experimental and computational methodologies, EXAFS simulations performed using FEFF, atomic model scaffold structures considered in this study, linear regression fitting of theoretical compounds, simulated XAS for Fe in diverse environments, XES data analysis procedures, computed energetics of Fe in various oxidation states and NiO_y_H_z_ scaffolds, and sample characterization information (PDF)
