# Supplementary material for: Operando Characterization of Fe in Doped Nix(Fe1–x)OyHz Catalysts for Electrochemical Oxygen Evolution
Source: J Am Chem Soc. 2025 Jan 25;147(5):4120–34. doi: 10.1021/jacs.4c13417 (PMC11803719; doi:10.1021/jacs.4c13417)
Supplement: Supplementary file 1 — ja4c13417_si_001.pdf [file ja4c13417_si_001.pdf]

Supporting information for:

Operando Characterization of Fe in Doped $Ni_x(Fe_{1-x})O_yH_z$ Catalysts for electrochemical oxygen evolution

Joakim Halldin Stenlid[1,2,3,$], Mikaela Görlin[1,4,$], Oscar Diaz-Morales[1,5], Bernadette Davies[1], Vladimir Grigorev[1,9], David Degerman[1,9], Aleksandr Kalinko,[6,7], Mia Börner[1], Mikhail Shipilin[1], Matthias Bauer,[6] Alessandro Gallo[2,3,8], Frank Abild-Pedersen[2], Michal Bajdich[2], Anders Nilsson[1,2,9] and Sergey Koroidov[1,9*]

[1] *Department of Physics, Alba Nova Research Center, Stockholm University, Stockholm, SE-106 91 Sweden.*

[2] *SUNCAT Center for Interface Science and Catalysis, SLAC National Accelerator Laboratory, 2575 Sandhill Road, Menlo Park, California, 94025, United States.*

[3] *SUNCAT Center for Interface Science and Catalysis, Department of Engineering, Stanford University, 443 Via Ortega, Stanford, California, 94305, United States.*

[4] *Department of Chemistry, Ångström Laboratory, Uppsala University, Uppsala, SE-751 21, Sweden*

[5] *Holst Centre, Netherlands Organisation for Applied Scientific Research, HighTech Campus 31, Eindhoven, 5656, The Netherlands.*

[6] *Department of Chemistry and Center for Sustainable Systems Design (CSSD), University of Paderborn, Warburger Strasse 100, Paderborn, D-33098, Germany*

[7] *Deutsches Elektronen-Synchrotron DESY, Notkestraße 85, Hamburg, D-22607, Germany.*

[8] *Sila Nanotechnologies, 2470 Mariner Square Loop, Alameda, California 94501, United States.*

[9] *Wallenberg Initiative Materials Science for Sustainability (WISE), Department of Physics, Stockholm University, Stockholm, SE-106 91, Sweden*

[$] Equal contributions

Corresponding Author[*] Email: sergey.koroidov@fysik.su.se

Contents

S1. Methods 2

S2. EXAFS simulations using FEFF 7

S3. Atomic models of NiO_yH_z scaffold structures considered in this work 16

S4. Linear regression fitting of theoretical compounds 18

S5. Total Fluorescence Yield vs High-energy resolution fluorescence detected 25

S6. Simulated XAS for Fe in varied environments 26

S7. XES data analysis details 28

S8. Computed energetics of Fe in varied oxidation states and NiOyHz scaffolds 30

S9. References 35

S1. Methods

S.1.1. Electrochemistry sample preparation and measurements

Following the same synthesis protocol as in Görlin et al.,[1] $Ni_x(Fe_{1-x})O_yH_z$ catalyst was prepared by electrodeposition on 10 x 10 mm graphene tape sheets (25 µm thick, Graphene Supermarket). Graphene sheets were used due to the flexibility of the material that benefits mechanical contact. Prior to the measurements, the surface of the graphene sheet was roughened with sand paper to improve the adhesion of the film. The deposition solution was prepared from 50 mM solutions of $Ni(NO_3)_2 \cdot 6H_2O$ (99.999% trace metals basis, Sigma-Aldrich) and $Fe(NO_3)_3 \cdot 9H_2O$ (≥99.999% trace metals basis, Sigma-Aldrich), mixed to a Ni:Fe molar ratio of 9:1. The catalyst film was deposited galvanostatically at a current density of -2.5 mA cm^{-2} for 113 s onto a geometric area of ~0.4 cm^2 in a 2-electrode setup employing a Pt-coil as counter electrode. A Bio-Logic SP-200 potentiostat was used to control the measurements. ICP-OES confirmed a Ni:Fe stoichiometry of 65:35 at. % in the final film, and a total metal loading of ~25 ± 2 µg cm^{-2}. The discrepancy between the molar ratio in the deposition solution and the final sample depends on differences in the deposition rates of Ni and Fe.

The electrochemical experiments were performed in a custom-made single compartment cell in a three-electrode configuration (see Figure 1): a leak-free Ag/AgCl (Harvard Instruments) reference electrode was used, a platinum mesh served as a counter electrode, and a $Ni_x(Fe_{1-x})O_yH_z$ on graphene sheets was used as working electrode (see above for preparation details of the working electrode). All measurements were controlled using a Bio-Logic SP-200 potentiostat. All the spectra (HERFD/EXAFS/XES) were collected at different potentials; starting and return to 1.1 V vs. RHE.

The electrochemical cell was installed at the angle of ~45° (to the surface normal) so that both the incident beam and the fluorescence penetrated through the working electrode to avoid interaction with the electrolyte.

All results are reproduced on three different samples prepared separately using the same synthesis protocol. Where applicable, the results are consistent with results using other electrolytes with varied composition including variation of the cation character.[1]

SEM analysis of the Ni–Fe catalyst post-OER revealed no significant changes in its morphology (Fig. S16). Energy-dispersive X-ray spectroscopy (EDS) further supports a non-covalent interaction between the catalyst and cations, as no traces of cations were detected in the Ni–Fe films post-OER following thorough rinsing with MilliQ water (Fig. S17a, b). In contrast, occasional traces of alkali cations were observed in films that were not rinsed as rigorously, likely originating from residual dried hydroxide salts. The Ni–Fe catalyst films exhibited notable local inhomogeneity in the Ni:Fe composition, with specific regions displaying clear Fe enrichment (Fig. S17c, d and Table S6). Such compositional variations are consistent with previous reports for Ni–Fe catalysts with Fe contents exceeding ~25%.[2,3] The presence of these variations in as-deposited films suggests they are unrelated to any specific alkali cation. Notably, the X-ray beam used for analysis is larger than the area shown in

Fig. S17d, indicating that the reported X-ray spectra represent an average across the total scanned region. Additionally, electrolyte impurities, such as Fe, could potentially incorporate into surface sites, influencing the catalytic activity and redox-peak behavior of Ni-based catalysts. To investigate this, ICP-OES and EDS analyses were performed to assess the purity of the alkali hydroxides. No detectable impurities were found in the purified hydroxides, confirming the success of the purification process (Fig. S18).

S.1.2. X-ray spectroscopy

All the X-ray spectroscopy measurements were performed at beamline P64 located at PETRA III[4] at DESY, Hamburg, Germany.

The incident energy was selected using a double-crystal monochromator with Si(311) or Si(111) crystals for the measurements, leading to a photon flux of approximately 10^{13} photons·s^{-1} on the sample position at 7112 keV and a beam size of about 70 µm × 200 µm (V × H). Incident energy calibration was performed using Fe and Ni foils. The first inflection point was used to find XAS Fe- and Ni K-edges positions at 7112 eV and 8333 eV respectively. XES and HERFD data were collected with a Von-Hamos-type spectrometer mounted in a Bragg scattering configuration. To record Fe and Ni Kα HERFD as well as Fe and Ni K_β XES the Von-Hamos spectrometer containing 8 cylindrically bent crystal analyzers was aligned to the corresponding emission lines, respectively.[5]

Energy calibration of the two-dimensional detector images was performed using elastic lines which span the spectrometer energy window for both Kα HERFD XAS and K_β XES. HERFD XAS spectra were generated as averaged intensities of the slice cut from RXES maps. The energetic width of the slice on the emitted energy axis (HERFD linewidth) was 1 eV and the cut was done through the RXES maximum. The non-resonant, incident energy RXES map was used to generate Ni and Fe Kα XAS spectrum. The combined resolution of the spectrometer and monochromator was determined to be 1.0eV for all the measurements.

A conventional fluorescence detector was used for EXAFS data collection with a passivated implanted planar silicon (PIPS) detector (Canberra).

All the spectra presented in this study correspond to the average of at least five repeats to achieve a satisfying signal-to-noise ratio. Radiation damage was assessed by monitoring spectral changes upon successive scans at one sample position, and no damage was observed.

Due to the high photon energies of the incident X-ray beam (hard X-ray), the measurements are bulk sensitive probing the entire material.

S.1.3. Computational models

Structural models for Fe-doped NiO_yH_z were created from pure NiO_yH_z layered structures of different formal oxidation state reported in the literature.[1,6–8] The Fe-doped but non-intercalated α-NiO_2, β-NiOOH, and β-$Ni(OH)_2$ phases where represented by three categories of models: i) Fe-rich models

with Fe replacing every forth Ni (i.e., 25%), and ii) Fe-poor models with a 6% Fe content – both categories comprising periodic models with three repeating periodic $Ni_x(Fe_{1-x})O_yH_z$ layers (in the *c*-direction) per unit cell. The Fe-rich models most closely represent the average experimental stoichiometry, whereas the Fe-poor models allow for examination of the structural effects of different NiO_yH_z scaffolds in the dilute limit. A third type of models, iii), with single layer $Ni_x(Fe_{1-x})O_yH_z$ structures cut out from the i) models were used in the XAS simulations as larger models become prohibitively expensive for these simulations whereas the spectral features are sufficiently modelled by the local environment captured in a single layer. Square pyramidal Fe coordination was simulated in the γ-NiOOH models, , starting from the *O*h structures and evaluating structures with one O/OH ligand removed.

Models of category i) have a 2×2×3 super cell size (i.e., 12 repeating $Ni_x(Fe_{1-x})O_yH_z$ units), and models ii) and iii) are of the 4×4×3 size in which two atomic layers have been deleted for the single layer models of category iii).

The intercalated α-$Ni(OH)_2$ and γ-NiOOH phases were modeled based on structures reported by Dionigi et al.[6] and Görlin et al.[1] with the layered structures impregnated with an aqueous NO^{3-} and K^+ layer for the α-$Ni(OH)_2$ (4×2×1 cell size) and γ-NiOOH (3×2×2) phase, respectively.

In all structures, intercalated or not, Fe doping was considered by replacing Ni with Fe in all unique positions but limited to one Fe per NiO_yH_z layer. The local structure around the Fe site was varied by changing x in FeO_6H_x from 0 to 6 in the original octahedral coordination, and additionally, for the single layer models, by comparing octahedral to square pyramidal and tetrahedral (T_d) coordination, as well as by replacing the axial O/OH ligands with water (H_2O-), peroxide (–OO, and –OO–), or oxo (=O) ligands. The H_2(g) reference state was modelled in a 21×22×23 $Å^3$ large cell (using a Γ-only **k**-mesh). All optimized structures are uploaded to the catalysis-hub repository[9] via https://www.catalysis-hub.org/publications/StenlidOperando2025 .

S.1.4. DFT calculations

Periodic DFT calculations were carried out with the Vienna Ab-initio Simulation Package (VASP, version 5.4.4).[10,11] The Perdew–Burke-Ernzerhof (PBE)[12] exchange-correlation functional was employed with Hubbard +*U* corrections[13] (Fe: 4.3, Ni: 3.0) where the *U-j* values compromise energetics between different oxidation states.[14] Core states were represented by projector augmented wave (PAW) potentials[15,16] and the (extended) valence states (Ni: $3d^9 4s^1$; Fe: $3d^7 4s^1$; K: $3p^6 4s^1$; O: $2s^2 2p^4$; N: $2s^2 2p^3$; H: $1s^1$) expanded on plane-wave basis sets with a 600 eV cut-off. The **k**-point grids considered for sampling the Brillouin zone were 6×6×4, 3×3×4, and 3×3×1 for the i), ii), and iii) models, respectively, whereas a 3×6×6 **k**-grid was used for α-$Ni(OH)_2$ and (4×6×3) for γ-NiOOH. Gaussian smearing was employed with a sigma value of 0.05 eV. The electronic and geometric convergence criteria were set to 10^{-5} eV and 10^{-2} eV/Å, respectively. The volume, shape, and ionic positions where optimized step-wise until convergence was met for the supercell structures. Spin-polarization was considered throughout.

Fe was modeled in a high-spin state in all cases based on the experimental data; tests conducted on Fe in the +2.00, +3.00 and +4.00 oxidation state in different NiO_yH_z scaffolds confirms that the high-spin state is the most stable configuration. Both ferromagnetic (FM) and antiferromagnetic (AFM) spin arrangements were evaluated (see discussion). Thermal corrections to Gibbs free energies at standard state were computed by numerical vibrational analysis under the harmonic oscillator and central difference approximations by ±0.015 Å displacements around the equilibrium positions. For the H_2 gas phase molecule, translational and rotational contributions were also added via the ideal gas and rigid rotor approximations. The computational hydrogen electrode (CHE) method was employed to account for the electrode potential.[17]

S.1.5. HERFD-XAS pre-edge simulations

Fe 1*s* XAS spectra with focus on the pre-edge structure were simulated using super-cell core-hole calculations in VASP (version 6.2.0) on the optimized structures, and compared to results from OCEAN 2.5.2 code[18,19] (https://github.com/times-software/OCEAN) that combines ground-state DFT calculations and the Bethe-Salpeter equation (BSE) to properly account for the presence of a core hole and the spin-orbit interactions of the metal centers. The Quantum Espresso (version 6.3)[20] electronic structure simulation package was used to obtain the DFT charge-density and wavefunctions. Norm-conserving pseudopotentials from the ABINIT88[21] distribution were used. A 4×4×1 **k**-point grid was employed for the DFT calculations and for the core-hole screening. The number of unoccupied bands used for the BSE and core-hole screening were approximately 200 and 1800, respectively. Both dipole and quadrupole transitions were considered following the procedure of Juhin et al.[22] A spectral broadening of 1.4 eV was applied and the spectra aligned with the onset of the white-line edge (7118 eV) and normalized by the area under the edge and high-energy post-edge fine-structure between 7118-7200 eV.

S.1.6. XANES and EXAFS simulations

The FEFF9 software[23] was used to simulate the Fe main peak XANES position and the EXAFS fine-structure details.

XANES: By using the ”COREHOLE none” card, potential and phase shift were calculated assuming complete screening of the core-hole, resulting in better agreement with experimental Fe K-edge XANES. The “SCF” radius for all calculations was set to R_{SCF}= 4 Å to include at least one surface unit cell. A “FMS” radius of 6 Å was found to be optimal to prevent scattering paths from being neglected and to ensure convergence of the multiple-scattering calculations for both structures.

EXAFS: The simulations were carried out in k space between 15-295 eV above E0 (k-range of 2-9 $Å^{-1}$). The values of E_0 were 8334 eV for the Ni K-edge and 7117 eV for the Fe K-edge. Due to the narrow k-range in this study, we considered only two shells (M-O and M-M coordination) in the EXAFS simulations. The Debye-Waller parameters (σ) were fixed at reasonable values for the respective shells

and the coordination numbers (CN), and absorber-scatterer distances (R) were minimized in the fit. The amplitude reduction factor (S02) was fixed at 0.85 for both Ni and Fe shells. The atom in access in our material (Ni) was selected as the scatterer both at the Ni and Fe K-edges, since the difference between using either Ni or Fe phase functions is anyhow not significant within the selected k-range (~1-9 $Å^{-1}$). The Levenberg-Marquardt algorithm with a 68 % confidence interval was employed. To extract oxidation states, calibration curves were constructed from known reference compounds (Supplementary Fig. 14).

S2. EXAFS simulations using FEFF

The k^3-weighted EXAFS spectra were extracted using an energy correction factor (E_0) of 8333 eV for the Ni *K*-edge and 7117 eV for the Fe *K*-edge. These were then minimized in the simulations for each applied potential (E_{app}). The XAS data was recorded up to a *k*-space of 9 Å^{-1}, and the simulations were performed between 2-9 Å^{-1} (15-300 eV above E_0). Scattering functions were generated in FEFF9 with the self-consistent field option switched on. The amplitude reduction factor (S_0^2) was set to 0.85 for both the Ni and Fe *K*-edges. The Debye-Waller parameters (DWs, σ) for the respective shells were obtained from the non-catalytic state (1.1 V_{RHE}), where the coordination numbers (CNs) were fixed at 6 (the expected values in a perfect crystal structure), and the DWs and the coordination distances (R) were minimized. For the consecutive OER catalytic potential (1.66 V_{RHE}), the DWs were fixed to the values obtained at 1.1 V_{RHE}, and the CNs (and R) were minimized. This approach allows for an estimate of the relative changes in coordination numbers as a response to the applied potential, which assumes that the DWs do not change with the applied potential, or the time exposed to the electrolyte. It was not possible to let both the DW and the CN free in the simulations simultaneously, whereby one of these parameters were restricted to avoid overparameterization. The longer Ni-O or Fe-O (2) shells appearing at ~3.5-3.7 Å originate from a real distance present in the crystal structure, which has a more significant contribution to the 2nd peak when the spectra are recorded to such short k-ranges. This longer M-O shell was therefore included in the simulations to yield a better fit. The contribution of the M-O peak is on the other hand minor in comparison to the contribution of the M-M shell. The oxidation states were determined both from the derived M-O coordination distances and from the K-edge positions at half-height of the normalized edge jump. The electrochemical measurements were carried out in purified 0.1 M KOH.

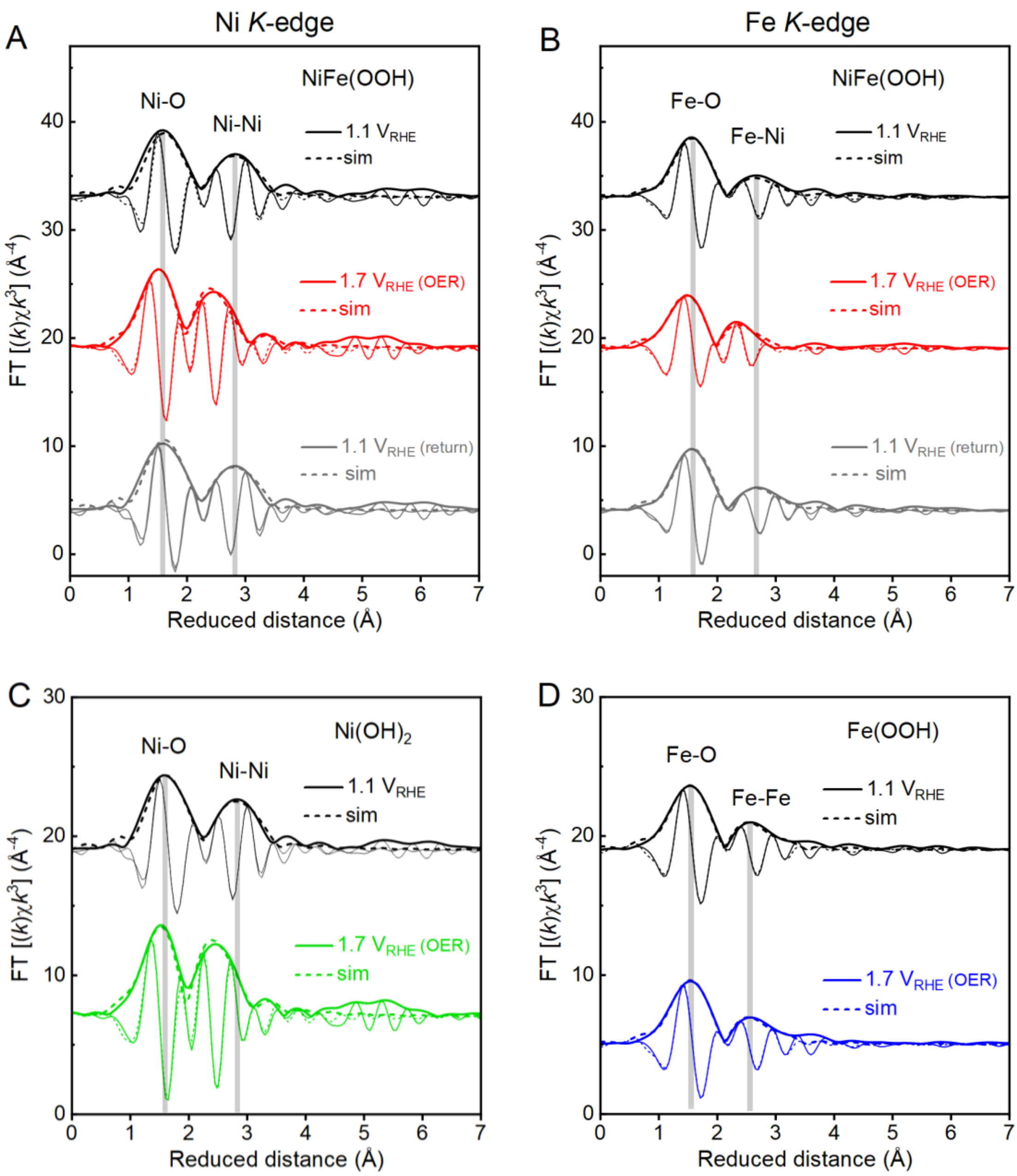

Figure S1. Fourier transformed k^3-weighted EXAFS of (A) $Ni(Fe)O_yH_z$ catalyst on the Ni *K*-edge. (B) $Ni(Fe)O_yH_z$ catalyst on the Fe *K*-edge. (C) $Ni(OH)_2$ catalyst on the Ni *K*-edge. (D) Fe(OOH) catalyst on the Fe *K*-edge. The measurements were carried out in purified 0.1 M KOH. The experimental spectra are shown as solid curves and the simulated spectra as dotted curves. The real part of the EXAFS is shown below the curves with the same color codes for experimental and simulated data. The gray shaded drop lines are added to guide the eye. The spectra were up to a k-range of 9 $Å^{-1}$ using a PIPS detector.

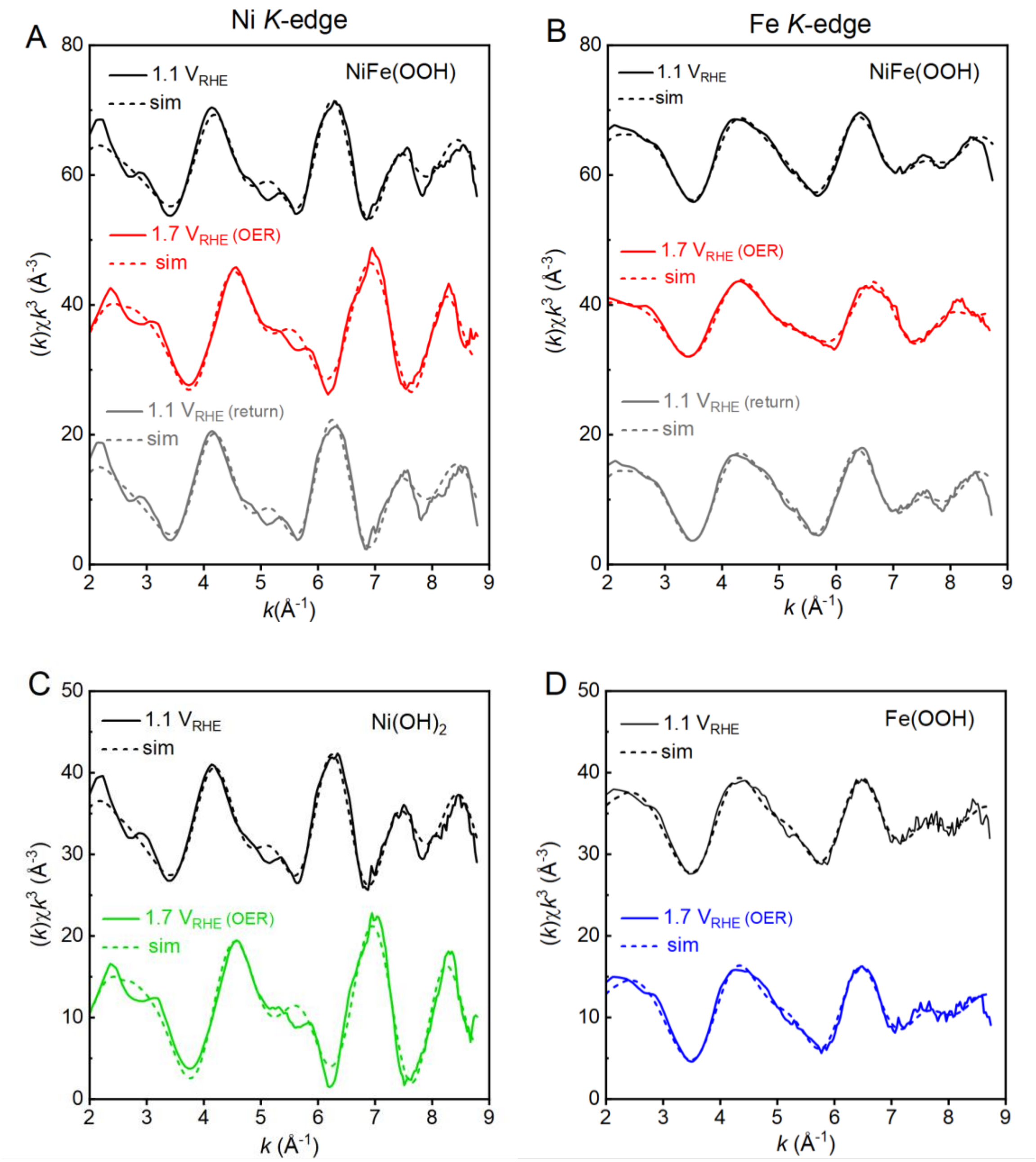

Figure S2. The k^3-weighted EXAFS χ(k) oscillations of the following: (a) Ni *K*-edge of the NiFe(OOH) catalyst. (b) Fe *K*-edge of the Ni(Fe)O_yH_z catalyst. (c) Ni *K*-edge of the pure NiO_yH_z catalyst. (d) Fe *K*-edge of the pure FeO_yH_z catalyst. The measurements were carried out in purified 0.1 M KOH. The experimental spectra are shown as solid curves and the simulated spectra as dotted curves. The spectra were up to a k-range of 9 $Å^{-1}$ using a PIPS detector.

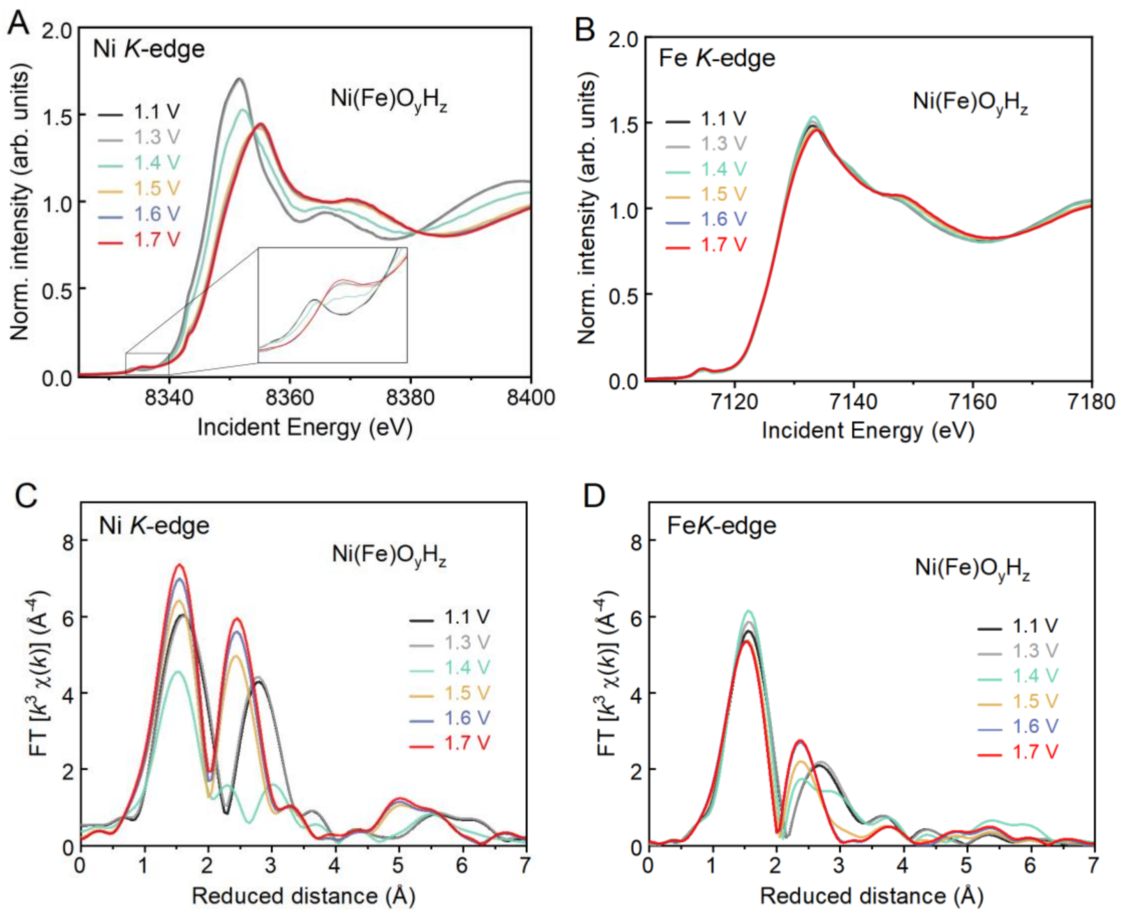

Figure S3. k^3 weighted XAS spectra of the $Ni(Fe)O_yH_z$ catalyst at various electrochemical potentials (1.1, 1.3, 1.4, 1.5, 1.6, and 1.7 V_{RHE}) in 0.1 M KOH. (a) The XANES at the Ni *K*-edge. (b) The XANES at the Fe *K*-edge. (c) FT-EXAFS at the Ni *K*-edge. (d) FT-EXAFS at the Fe *K*-edge. The asterisk in (a) marks a glitch in the rising edge presumably from the crystal in the monochromator. The spectra were recorded up to a k-range of 9 $Å^{-1}$ using a PIPS detector.

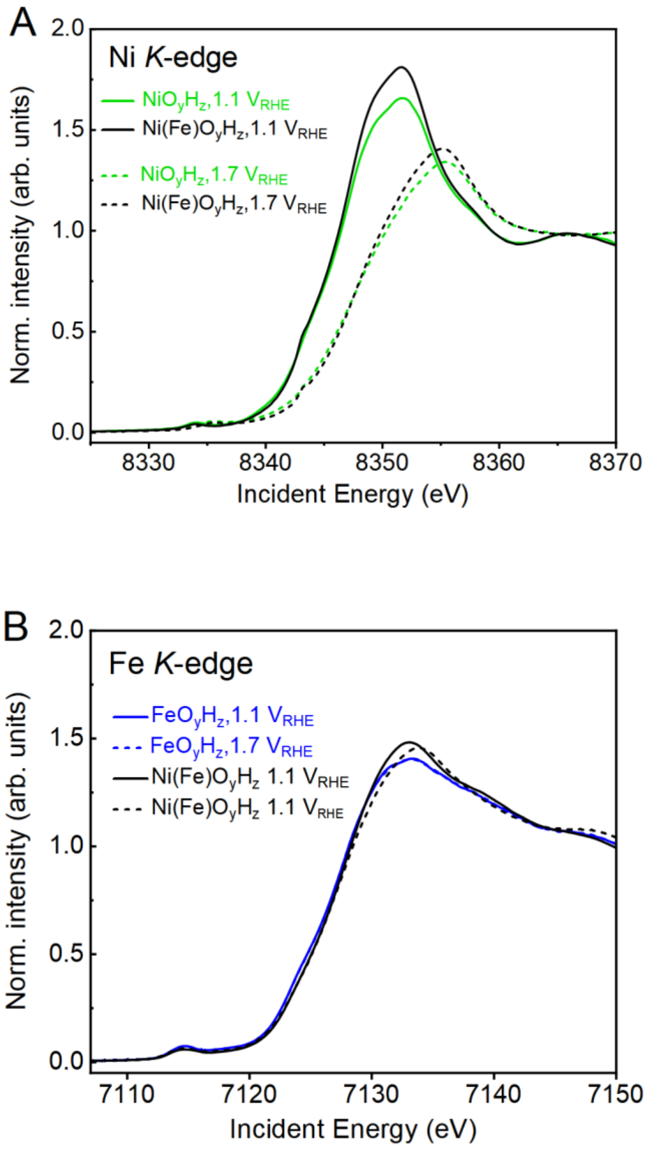

Figure S4. XANES spectra at the (a) Ni *K*-edge (b) Fe *K*-edge comparing the Ni(Fe)O_yH_z catalyst with the pure NiO_zH_y and FeO_zH_y catalysts. The spectra were measured up to a k-range of 9 Å^{-1} using a PIPS detector.

Table S1. Experimental fit parameters of the Ni(Fe)O_yH_z catalyst at the Ni and Fe *K*-edges. The data was measured using a PIPS detector between a k-range of 2-9 $Å^{-1}$. The Debye-Waller parameters (DW, σ) were determined from the data at 1.1 V_{RHE} by keeping the coordination numbers (CN) fixed to 6. For the following potentials, the CNs were minimized in the simulations. The fit was carried out using a 68 % confidence interval, where the R_f factor represents the difference between the simulated and the experimental spectra.

**Ni *K*-edge**	**R (Å)**	**CN**	**σ (Å)**	**ΔE_0**	**R_f (%)**
E ≈ 1.1 V_{RHE} (non-catalytic)					
Ni-O (1st)	2.035 ± 0.007	6 [1)]	0.090	2.2	18.9
Ni-Ni	3.079 ± 0.007	6 [1)]	0.098		
Ni-O (2nd)	3.61 ± 0.03	6 [1)]	0.090 [2)]		
E ≈ 1.7 V_{RHE} (OER catalytic)					
Ni-O (1st)	1.888 ± 0.006	5.3 ± 0.2	0.090 [2)]	3.9	17.4
Ni-Ni	2.823 ± 0.005	7.0 ± 0.3	0.098 [2)]		
Ni-O (2nd)	3.53 ± 0.03	4.2 ± 1.1	0.090 [2)]		
E ≈ 1.1 V_{RHE} return					
Ni-O (1st)	2.041 ± 0.007	6.3 ± 0.2	0.090 [2)]	2.7	18.4
Ni-Ni	3.082 ± 0.007	6.5 ± 0.5	0.098 [2)]		
Ni-O (2nd)	3.63 ± 0.03	6.3 ± 1.3	0.090 [2)]		
**Fe *K*-edge**	**R (Å)**	**CN**	**σ (Å)**	**ΔE_0**	**R_f**
E ≈ 1.1 V_{RHE} (non-catalytic)					
Fe-O (1st)	1.985 ± 0.008	6 [1)]	0.106	1.5	11.2
Fe-Ni	3.050 ± 0.019	6 [1)]	0.149		
Fe-O (2nd)	3.52 ± 0.03	6 [1)]	0.106 [2)]		
E ≈ 1.7 V_{RHE} (OER catalytic)					
Fe-O (1st)	1.938 ± 0.013	5.3 ± 0.4	0.106 [2)]	-4.3	15.2
Fe-Ni	2.889 ± 0.018	7.9 ± 1.4	0.149 [2)]		
Fe-O (2nd)	3.59 ± 0.07	3.1 ± 2.0	0.106 [2)]		
E ≈ 1.1 V_{RHE} return					
Fe-O (1st)	1.997 ± 0.008	6.1 ± 0.3	0.106 [2)]	1.6	10.6

Fe-Ni	3.075 ± 0.014	8.9 ± 1.3	0.149 [2)]
Fe-O (2nd)	3.52 ± 0.13	1.2 ± 1.4	0.106 [2)]

[1)] The CN for the E ≈ 1.1 V_{RHE} was fixed to 6 to allow for determination of the DW parameters (σ) for the consecutive potentials.

[2)] The σ parameter for the 2nd M-O shell was fixed to the corresponding value of the 1st M-O shell for the respective potential.

Table S2. Experimental fit parameters of the pure $Ni(OH)_2$ and FeOOH catalysts. The data was measured using a PIPS detector between a k-range of 2-9 $Å^{-1}$. The Debye-Waller parameters (DW, σ) were initially determined from the non-catalytic potential (1.1 V_{RHE}) for the respective shells by keeping the coordination numbers (CN) fixed to 6. The DWs were then kept fixed for the consecutive potentials while the CNs were minimized in the simulations. The fit parameters were carried out using a 68 % confidence interval, where the R_f factor represents the difference between the simulated and the experimental spectra.

**NiO_zH_y**	**R (Å)**	**CN**	**σ (Å)**	**ΔE_0 (eV)**	**R_f (%)**
E ≈ 1.1 V_{RHE} (non-catalytic)					
Ni-O (1st)	2.035 ± 0.008	6 [1)]	0.101	2.3	19.4
Ni-Ni	3.086 ± 0.007	6 [1)]	0.101		
Ni-O (2nd)	3.61 ± 0.03	6 [1)]	0.101 [2)]		
E ≈ 1.7 V_{RHE} (OER-catalytic)					
Ni-O (1st)	1.883 ± 0.009	5.0 ± 0.3	0.101 [2)]	4.1	22.9
Ni-Ni	2.810 ± 0.007	7.4 ± 0.5	0.101 [2)]		
Ni-O (2nd)	3.54 ± 0.05	3.9 ± 1.7	0.101 [2)]		
E ≈ 1.1 V_{RHE} return					
Ni-O (1st)	2.033 ± 0.008	5.9 ± 0.4	0.101 [2)]	0.9	17.5
Ni-Ni	3.084 ± 0.008	5.2 ± 0.7	0.101 [2)]		
Ni-O (2nd)	3.54 ± 0.03	5.9 ± 2.1	0.101 [2)]		
**FeOOH**	**R (Å)**	**CN**	**σ (Å)**	**ΔE_0 (eV)**	**R_f (%)**
E ≈ 1.1 V_{RHE} (non-catalytic)					
Fe-O	1.971 ± 0.010	6 [1)]	0.120	-0.4	14.5
Fe-Fe	3.010 ± 0.013	6 [1)]	0.139		
E ≈ 1.7 V_{RHE} (OER catalytic)					
Fe-O	1.975 ± 0.016	6.0 ± 0.5	0.120 [2)]	-4.0	16.4
Fe-Fe	3.013 ± 0.09	6.1 ± 1.2	0.139 [2)]		

1) The CN for the E ≈ 1.1 V_{RHE} was fixed to 6 to allow for determination of the DW parameters (σ) used for the consecutive potentials.

2) The σ parameter for the 2nd M-O shell was fixed to the corresponding value of the 1st M-O shell for the respective potential.

S3. Atomic models of NiO_yH_z scaffold structures considered in this work

Figure S5 summarizes the various NiO_yH_z scaffold structures used in this work, including both non-intercalated and intercalated structures. From these, different degrees of Fe-doping where considered as described in the main text.

To quantify the observed changes occurring at the different potentials applied, EXAFS fits using the FEFF multiple scattering approach were initially performed for both the Fe and Ni data on layered Fe-doped (25%; i.e., $Ni_3Fe_1O_yH_z$) non-intercalated NiO_2 scaffold structures. We omitted protons (H^+) as they will not affect the simulated spectra. These structures are representative of typical NiO_yH_z structures with varying symmetric and asymmetric bond contractions.

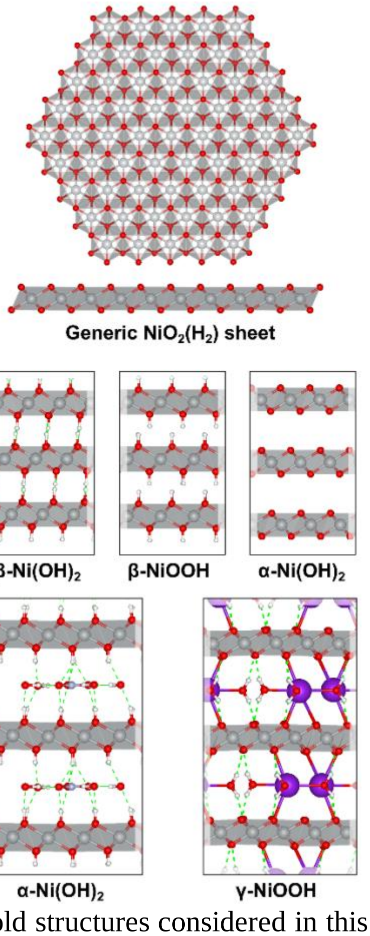

Figure S5. Atomic models of NiO_yH_z scaffold structures considered in this work. Top panel shows a generic NiO_2 sheet. This structure is the basis of all the considered NiO_yH_Z phases, which can be formed by stacking the NiO_2 sheets, (partially) protonating them, and through solvent and ion intercalation in between the layers. The middle panel contains non-intercalated structures of NiO_yH_z with Ni in varied oxidation state. The bottom panel displays intercalated structures.

We also considered intercalated structures, with approximately 10% Fe concentration. In the intercalated models, the space between the $Ni_x(Fe_{1-x})O_yH_z$ sheets is occupied by electrolyte ions and water molecules: in the case of α-$Ni(OH)_2$, the intercalated layer is formed by water and NO_3^- ions,

while for γ-NiOOH, the NO_3^- ions are replaced by K^+ ions. The results show that intercalation, especially with K^+, leads to local symmetry breaking and a larger structural variation within the material

S4. Linear regression fitting of theoretical compounds

S4.1. FEFF simulated DFT optimized theoretical structures

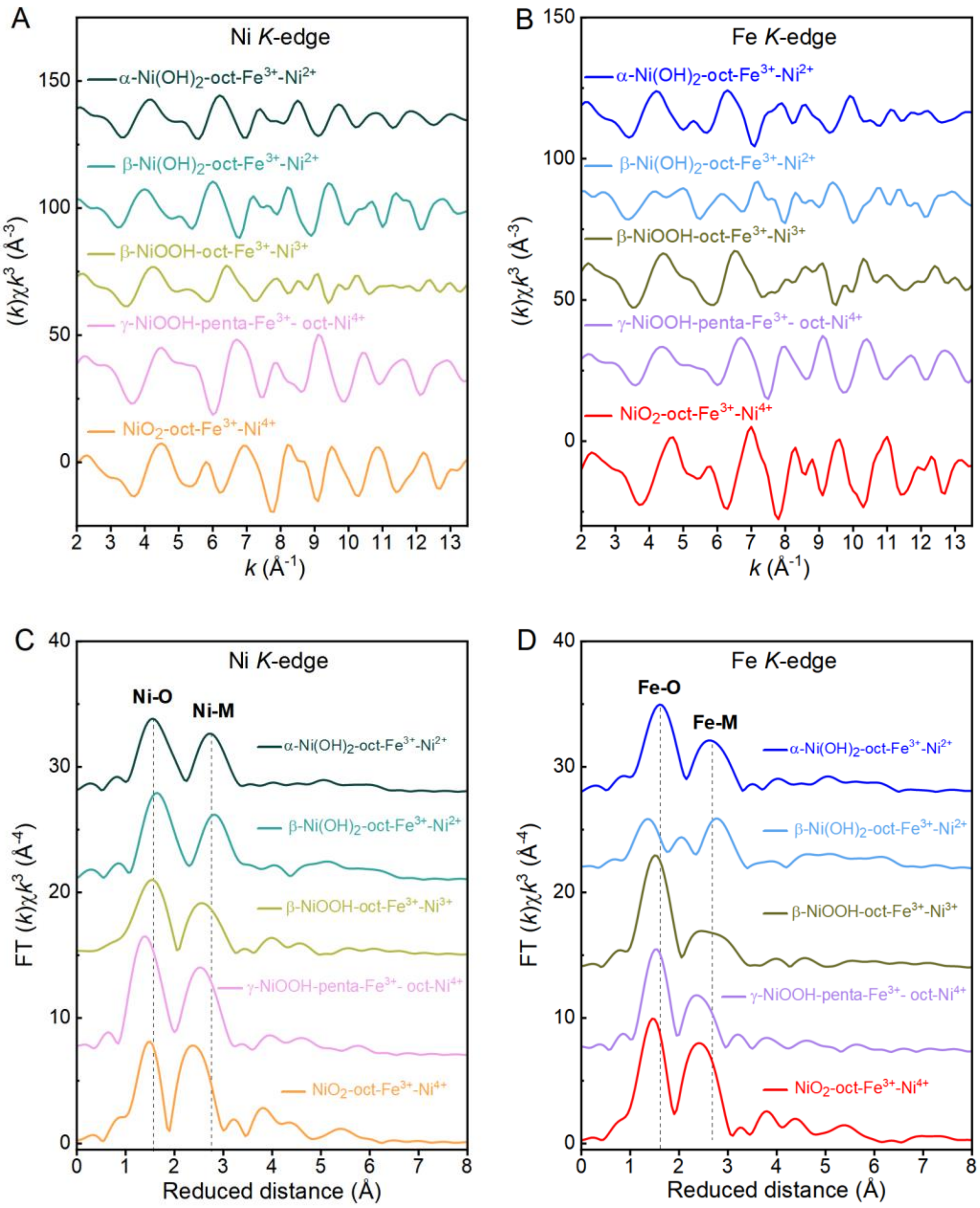

Figure S6. FEFF modeled k^3-weighted EXAFS and FT-EXAFS spectra of theoretical structures optimized using DFT. **(a)** k-space (chi) at the Ni *K*-edge **(b)** k-space (chi) at the Fe *K*-edge **(c)** FT-EXAFS at the Ni *K*-edge **(d)** FT-EXAFS at the Fe *K*-edge. The FT-EXAFS was simulated using an energy range of 15-300 eV (k-range of 1.98 – 8.87 $Å^{-1}$) to match the experimental spectra. Note that all structures are Fe-doped.

S4.2. Linear combination fitting

Experimental: The k^3 weighted EXAFS spectra of the DFT optimized theoretical structures were initially calculated using SimXLite, which implements the FEFF9 code. The FT-EXAFS spectra were modelled between a k-range of 1.98-8.87 $Å^{-1}$ (15-300 eV) to match the experimental data. Subsequently, linear combination fitting was performed to assess whether the experimental spectra could be accurately described by the DFT optimized structures. Two spectra of the electrodeposited NiFe catalyst were utilized: one at non-catalytic potential (1.1 V_{RHE}) and another one at OER-catalytic potential (1.7 V_{RHE}). Initially, more structures were screened including crystallographic information files (cif); however, only the best matches are presented. Some of the Fe-doped theoretical structures included various sites, both hexa-coordinated (i.e., octahedral) and penta-coordinated; only the best matches are shown.

Results: The results from the linear combination fitting are presented in Table S5 and Figures S6-S8. At the Ni K-edge, the experimental spectra at non-catalytic potential (1.1 V_{RHE}) show best match with the Fe-doped theoretical structures α-$Ni(OH)_2$, β-$Ni(OH)_2$, and β-NiOOH. At an applied OER catalytic potential (1.7 V_{RHE}), the Ni *K*-edge spectra are best described by the hexa-coordinated site (i.e., octahedral site) in the two oxidized structures γ-NiOOH and NiO_2. Note that the Ni *K*-edge only matches with octahedral sites. At the Fe K-edge, the experimental spectra at 1.1 V_{RHE} show the best match with the theoretical structures α-$Ni(OH)_2$, β-$Ni(OH)_2$, and β-NiOOH, similar to the results on the Ni *K*-edge. At an applied potential of 1.7 V_{RHE}, the Fe *K*-edge spectra are best described by a mixture of various structures; however, the highest scores are obtained for the penta-coordinated site in the γ-NiOOH structure and the octahedral site in the NiO_2 structure. The major differences between the Ni and Fe *K*-edges lie in the structure during applied OER catalytic potential, where the Fe-atoms preferably occupy penta-coordinated sites and Ni-atoms hexa-coordinated sites. The Fe *K*-edge also appears more disordered than the Ni *K*-edge, which may account for its matches with a wider range of structures.

Table S3. Local atomic parameters of the DFT optimized theoretical structures used in the linear combination fitting at Fe *K*-edge. The respective shells were grouped within 0.3 Å atomic radius.

**Structure/shell**	**R (Å)**	**CN**
**α-Ni(OH)$_2$-oct-Fe^{3+}Ni^{2+}**		
Fe-O	2.039	6
Fe-Ni	3.013	6
Fe-O	3.622	6
Fe-O	4.708	12
Fe-Ni	5.284	6
Fe-Ni	6.102	4
Fe-Fe	6.149	2
**β-Ni(OH)$_2$-oct- Fe^{3+}Ni^{2+}**		
Fe-O	1.989	6
Fe-Ni	3.143	6
Fe-O	3.759	6
Fe-O	4.966	12
Fe-Ni	5.449	6
Fe-Fe	6.292	6
**β-NiOOH-oct- Fe^{3+}Ni^{3+}**		
Fe-O	1.945	6
Fe-Ni	2.945	6
Fe-O	3.546	6
Fe-O	4.423	4
Fe-O	4.734	8
Fe-Ni	5.104	6
Fe-Fe	5.682	2
**γ-NiOOH-penta- Fe^{3+}-oct-Ni^{4+}**		
Fe-O	1.938	5
Fe-Ni	2.891	6
Fe-O	3.591	6
Fe-O	4.552	11
Fe-Fe	4.975	2
Fe-Ni	4.997	4
Fe-Ni	5.761	6
**NiO$_2$ oct- Fe^{3+}Ni^{4+}**		
Fe-O	1.872	6
Fe-Ni	2.768	6
Fe-O	3.339	6
Fe-O	4.357	12
Fe-Ni	4.769	4
Fe-Fe	4.779	2
Fe-Ni	5.521	4
Fe-Fe	5.530	2

Table S4. Local atomic parameters of the DFT optimized theoretical structures used in the linear combination fitting at Ni *K*-edge. The respective shells were grouped within 0.3 Å atomic radius.

**Structure/shell**	**R (Å)**	**CN**
**α-Ni(OH)$_2$-oct-Fe^{3+}Ni^{2+}**		
Ni-O	2.021	6
Ni-Fe	3.015	1
Ni-Ni	3.071	5
Ni-O	3.672	6
Ni-O	4.781	12
Ni-Fe	5.285	1
Ni-Ni	5.299	5
**β-Ni(OH)$_2$-oct- Fe^{3+}Ni^{2+}**		
Ni-O	2.094	6
Ni-Fe	3.143	2
Ni-Ni	3.146	4
Ni-O	3.763	6
Ni-O	4.884	12
Ni-Ni	5.449	4
Ni-Fe	5.449	2
**β-NiOOH-oct- Fe^{3+}Ni^{3+}**		
Ni-O	1.965	6
Ni-Ni	2.942	4
Ni-Fe	2.947	2
Ni-O	3.542	6
Ni-O	4.596	12
Ni-Fe	5.099	2
Ni-Ni	5.104	4
**γ-NiOOH-penta- Fe^{3+}-oct-Ni^{4+}**		
Ni-O	1.932	6
Ni-Ni	2.866	5
Ni-Fe	2.889	1
Ni-O	3.422	5
Ni-O	3.755	1
Ni-O	4.474	9
Ni-O	4.831	1
Ni-Ni	4.989	6
Ni-Fe	5.761	3
Ni-Ni	5.763	3
**NiO$_2$ oct- Fe^{3+}Ni^{4+}**		
Ni-O	1.899	6
Ni-Ni	2.755	4
Ni-Fe	2.769	2
Ni-O	3.352	6
Ni-O	4.335	12
Ni-Fe	4.771	2
Ni-Ni	4.793	4

Table *S5*. Linear combination of the electrodeposited NiFe catalyst by comparison to the DFT optimized theoretical structures. The linear combination was performed in k-space between 1.98 – 8.87 $Å^{-1}$ (15-300 eV). The results are reported in percentages, and the error is given in brackets. The goodness of fit was evaluated using the R-factor filtered value, listed at the bottom of the table. Only the best matches are shown; initially, more structures were screened. Note that all theoretical structures are Fe-doped; the coordination number for the Ni and Fe sites are indicated for each structure as either octa (hexa-coordinated) or penta-coordinated.

**Simulation range** ***k*** **(2-9 $Å^{-1}$)**	**Matching phases (%)**			
	**Ni K-edge**		**Fe K-edge**	
	**1.1 V_{RHE}**	**1.7 V_{RHE}**	**1.1 V_{RHE}**	**1.7 V_{RHE}**
α-$Ni(OH)_2$-oct-$Fe^{3+}Ni^{2+}$	54 (12)	0 (16)	38 (6)	11 (8)
β-$Ni(OH)_2$-oct- $Fe^{3+}Ni^{2+}$	19 (6)	0 (8)	30 (6)	12 (7)
β-NiOOH-oct- $Fe^{3+}Ni^{3+}$	27 (12)	0 (15)	27 (7)	26 (8)
γ-NiOOH-penta- Fe^{3+}-oct-Ni^{4+}	0 (8)	44 (11)	1 (6)	37 (6)
NiO_2 oct- $Fe^{3+}Ni^{4+}$	0 (4)	56 (6)	5 (4)	14 (6)
Sum	100	100	100	100
R-factor filtered	27.1	33.5	20.8	15.1

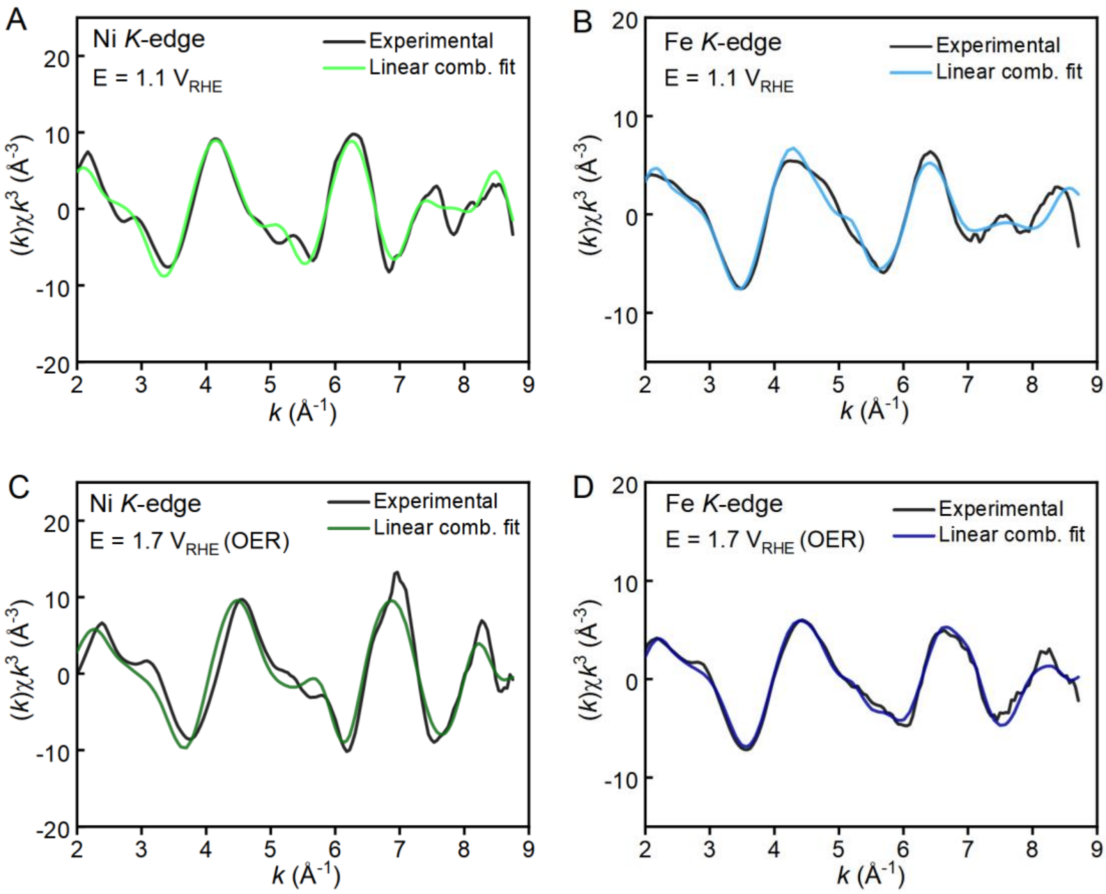

Figure S7. Linear combination fitting shown in k-space. Comparison of the experimental spectra of the NiFe catalyst and the linearly combined spectra (linear comb. fit) using various DFT optimized structures **(a)** Ni *K*-edge at 1.1 V (non-catalytic) **(b)** Ni *K*-edge at 1.7 V (OER-catalytic) **(c)** Fe *K*-edge at 1.1 V (non-catalytic) **(d)** Fe *K*-edge at 1.7 V (OER-catalytic).

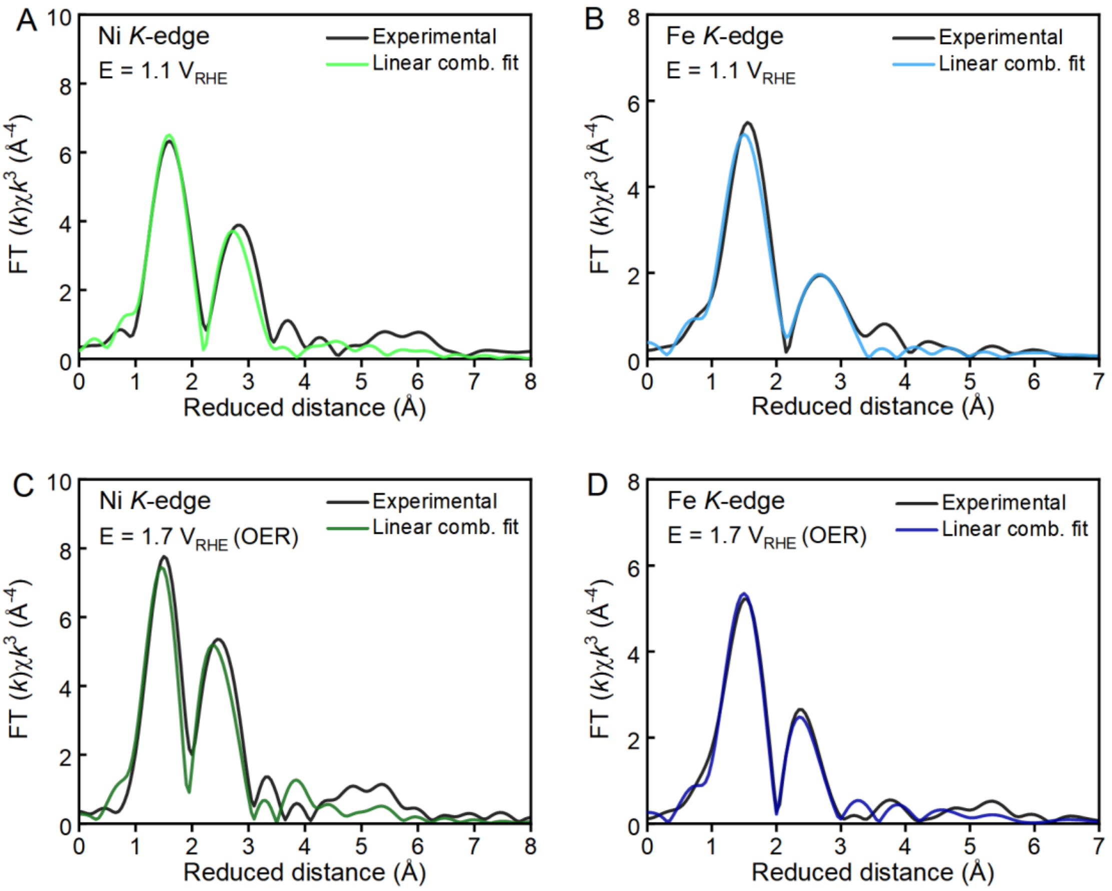

Figure S8. Linear combination fitting shown in R-space. Comparison of the experimental spectra of the NiFe catalyst and the linearly combined spectra (linear comb. fit) using various DFT optimized structures **(a)** Ni *K*-edge at 1.1 V (non-catalytic) **(b)** Ni *K*-edge at 1.7 V (OER-catalytic) **(c)** Fe *K*-edge at 1.1 V (non-catalytic) **(d)** Fe *K*-edge at 1.7 V (OER-catalytic).

S5. Total Fluorescence Yield vs High-energy resolution fluorescence detected

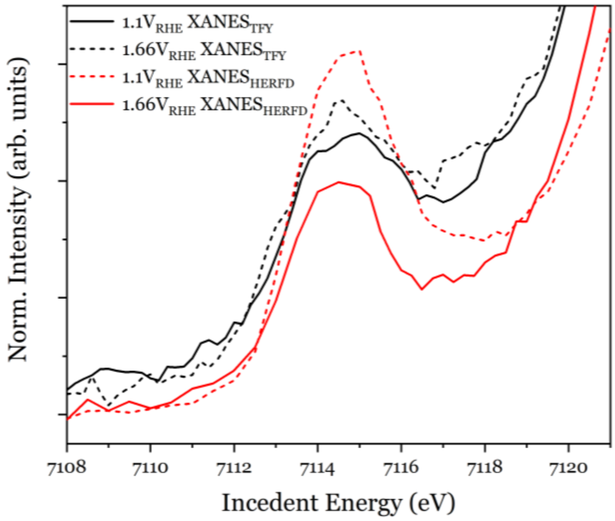

Figure S9. High-energy resolution fluorescence detected (HERFD) Fe K-edge XANES spectra of Ni(Fe)O_yH_z the resting (at 1.10 V_{RHE}) and operational (1.66 V_{RHE}). The spectra are compared with total fluorescence yield (TFY) curves recorded using a photodiode.

S6. Simulated XAS for Fe in varied environments

Shown in Figures S10-12 are DFT-simulated XAS spectra for Fe in different oxidation states, different NiO_yH_z scaffold structures, and in different ligand environments.

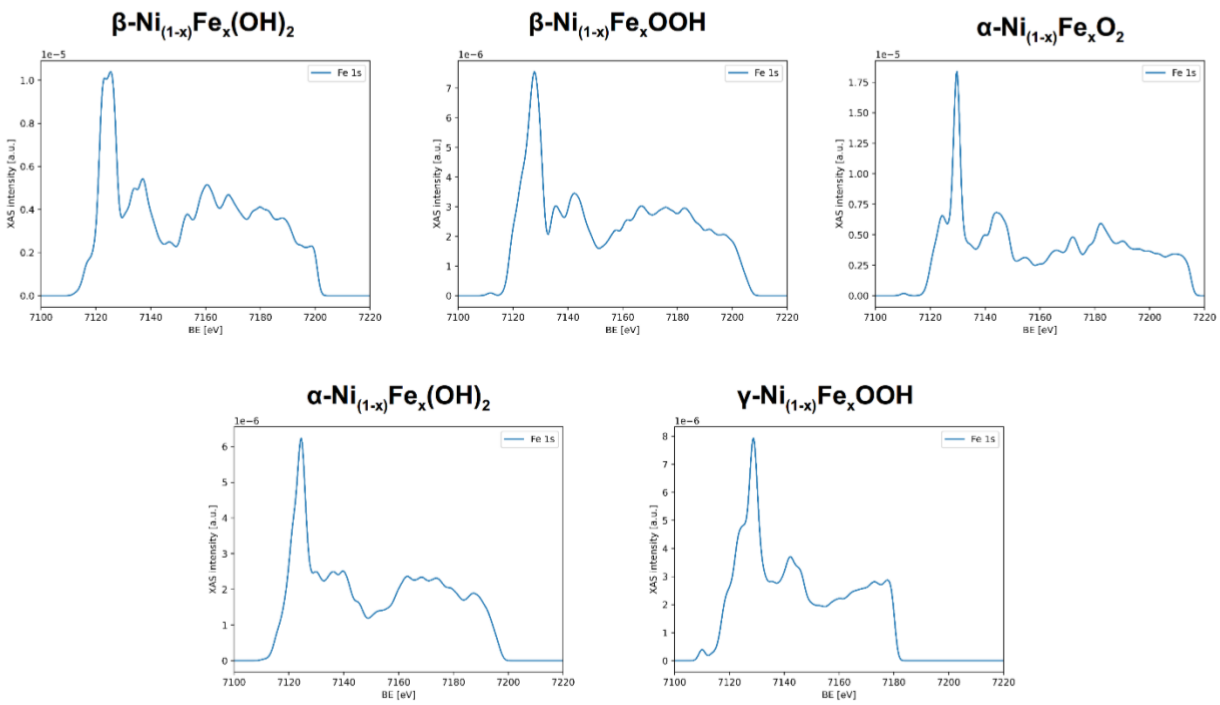

Figure S10. DFT-simulated Fe 1s XAS spectra for Fe in varied non-intercalated $Ni_{(x-1)}Fe_xO_yH_z$ scaffolds with Fe in oxidation states ranging from +2 to +4 depending on the scaffold structure. Fe:Ni ratio 1:12.

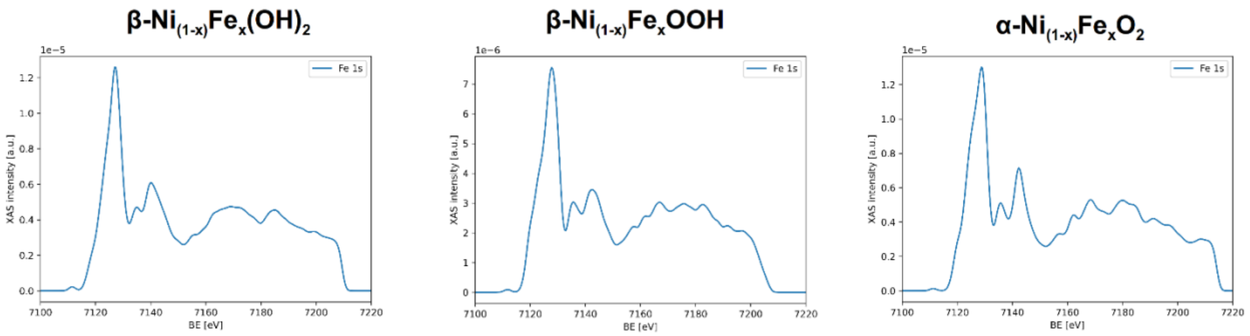

Figure S11. DFT-simulated Fe 1s XAS spectra for Fe in varied non-intercalated $Ni_{(x-1)}Fe_xO_yH_z$ scaffolds with a constant Fe(+3.00) oxidation state. Fe:Ni ratio 1:12.

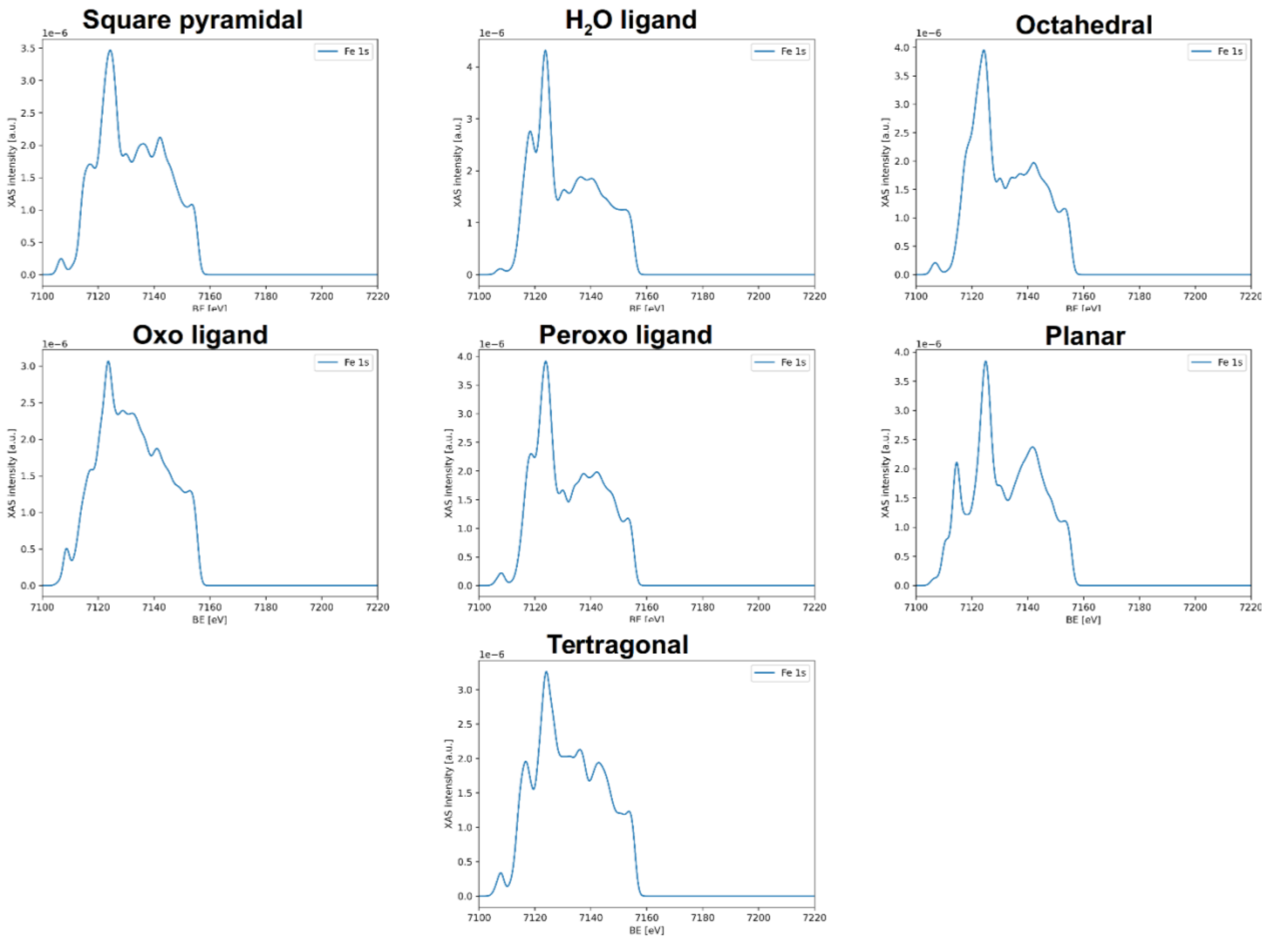

Figure S12. DFT-simulated Fe 1s XAS spectra for Fe in $Ni_{(x-1)}Fe_xOOH$ scaffold with varied ligand environment around the Fe(~+3.00) center. Fe:Ni ratio 1:12.

S7. XES data analysis details

XES spectra for both $K\alpha$ and $K\beta$ were area normalized using the full spectral range. The center of gravity (COG) parameter was defined as follows:

$$COG = \frac{\sum_i (E_i \cdot I_i)}{\sum_i (I_i)},$$

where E_i and I_i are the energy and intensity of the data point i, respectively. The COG determination is motivated by the suggestion made by P. Glatzel,[24,25] where it was proposed that to remove the variations due to screening effects, all the spectra to be compared should be aligned to have equal COG. In the case of $K\beta$, this should allow only observing changes due to the 3p-3d exchange interaction variations in the spectra.

The integrated absolute difference (IAD) was proposed for XES by Vankó et al.,[26] and the results successfully extract the spin evolution from the $K\beta$ emission spectra. The IAD is given by

$$IAD_j = \int_{E_n}^{E_f} |SP_{ox} - SP_{rest}| dE,$$

with SP_{ox} and SP_{rest} are the spectrum, rest as the resting state (here at 1.10 V_{RHE}), and an ox spectrum of the catalyst's state with the applied potential above the resting state, respectively (both normalized to unity area). This method provides a way to obtain information from the whole spectra (the integral is calculated over the whole energy range recorded, from the initial to the final point, E_i and E_f, respectively). IAD is a useful tool for rapidly determining the spin state, oxidation state change, or any variation in $K\beta$ XES spectra.

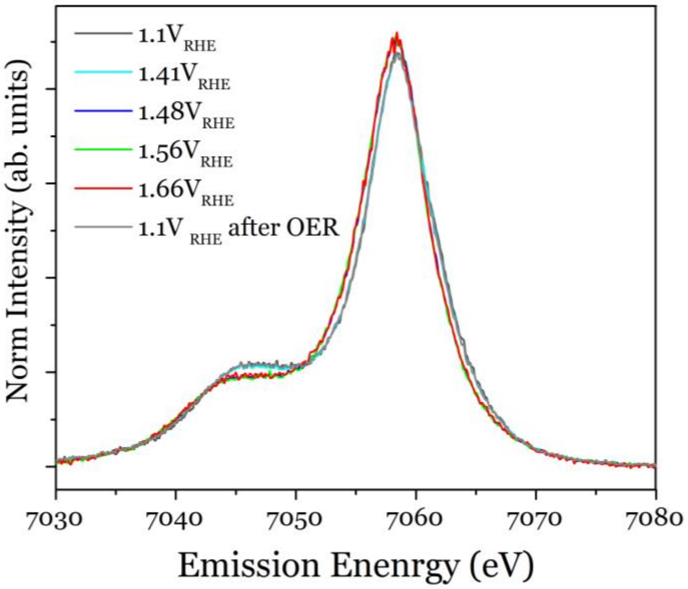

Figure S13. Fe Kβ XES spectra of $Ni(Fe)O_xH_y$ cycled from 1.10 V_{RHE} to 1.66 V_{RHE} and back to 1.10 V_{RHE} after OER

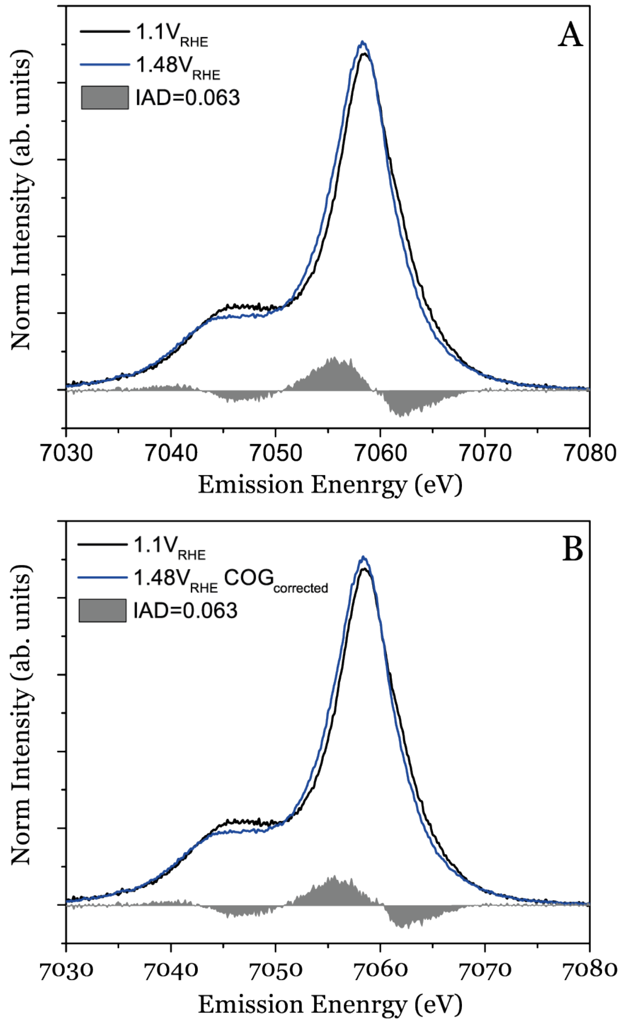

Figure S14. Difference spectra (grey dashed area) between Ni(Fe)O_xH_y at 1.10 V_{RHE} and 1.48 V_{RHE} before (A) and after (B) the COG alignment. The resulting IAD values are indicated in the figures

S8. Computed energetics of Fe in varied oxidation states and NiOyHz scaffolds

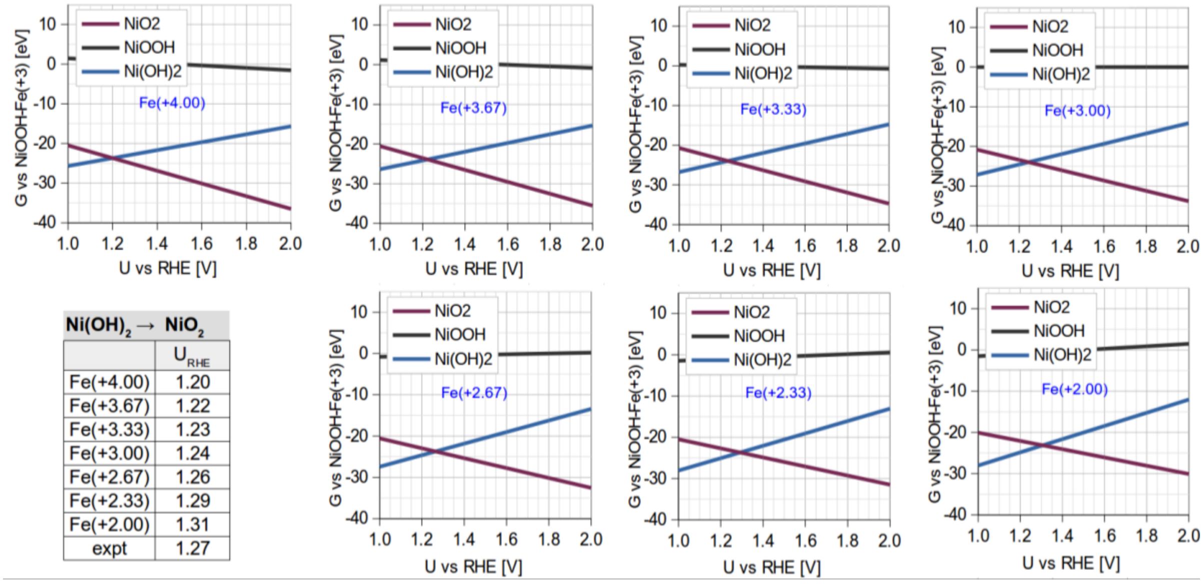

$Ni(OH)_2 \rightarrow NiO_2$	
	U_{RHE}
Fe(+4.00)	1.20
Fe(+3.67)	1.22
Fe(+3.33)	1.23
Fe(+3.00)	1.24
Fe(+2.67)	1.26
Fe(+2.33)	1.29
Fe(+2.00)	1.31
expt	1.27

Figure S15. Potential-dependent DFT computed Gibbs free energies of non-intercalated $Ni_xFe_{(1-x)}O_yH_z$ (x=15/16) with Fe in formal oxidation state ranging from +2.00 to +4.00. Unit cells comprising three layers and four transition metals (TM, i.e., Ni and Fe) per layer were used, with Fe substituting Ni in one of the 16 TM locations. Noticeably, $TM(OH)_2$ is favored at the resting state potential (1.10 V_{RHE}) and TMO_2 at the OER operating conditions (> 1.48 V_{RHE}) used in this study regardless of the oxidation state of Fe.

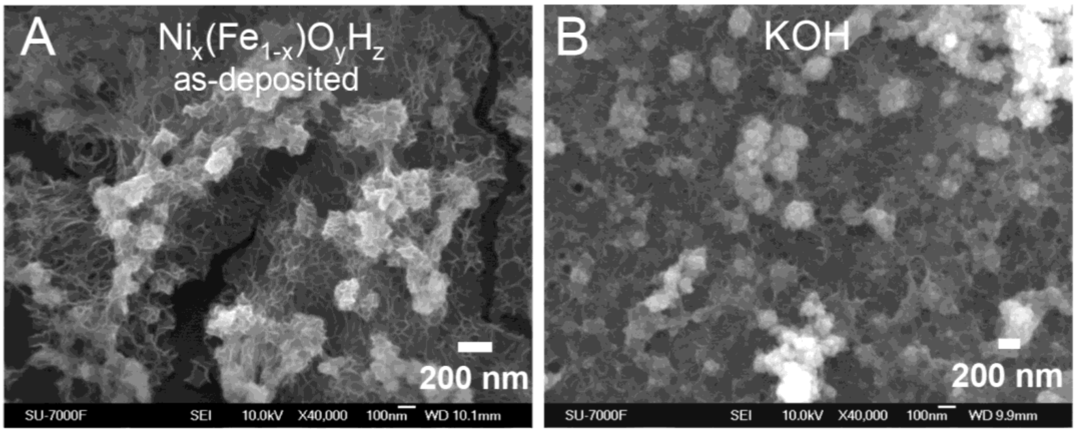

Figure S16. Scanning Electron Microscopy (SEM) of the electrodeposited $Ni_x(Fe_{1-x})O_yH_z$ catalyst on graphene electrode **(A)** as-deposited film not exposed to electrolyte, and films investigated post-OER after the in situ XAS measurements carried out in (B) 0.1 M KOH electrolytes

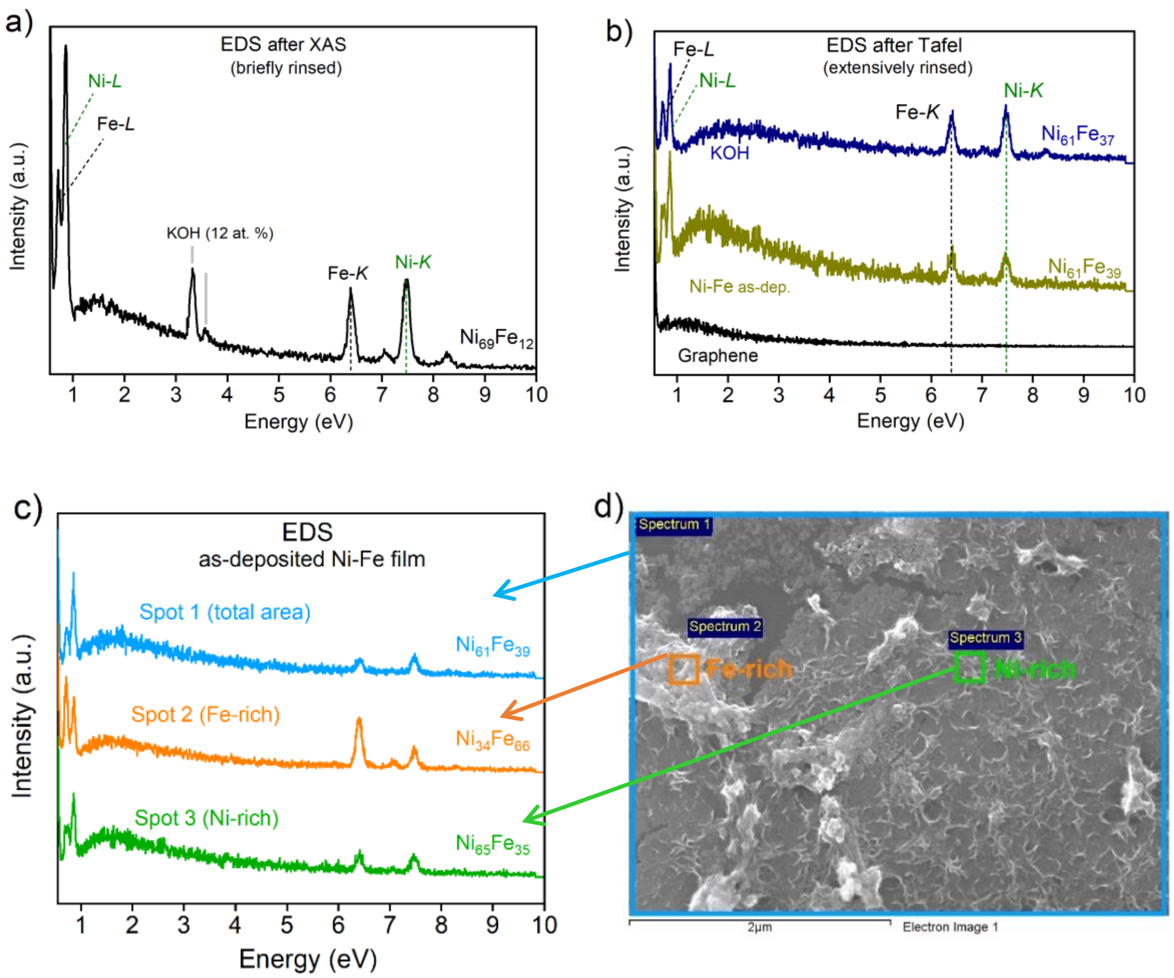

Figure S17. Energy dispersive X-ray spectroscopy (EDS) of the $Ni_x(Fe_{1-x})O_yH_z$ catalyst on graphene electrode after investigations in 0.1 M KOH (a) selected EDS spectra of sample area after the in situ XAS measurements. This sample was rinsed only briefly with Milli-Q water after the measurement (the rinsing step was not controlled), and traces of alkali metal cations were visible in the films in the EDS spectra, marked with drop-lines. The atomic percentages are given in brackets. **(b)** EDS spectra of selected sample areas after Tafel slope collection. These samples were rinsed extensively (and in a controlled manner) by dipping the films ca. five times in fresh Milli-Q water immediately after the measurements to remove non-specifically adsorbed salts before they dry. There were no traces of alkali metal cations detected in these films. The two lower EDS spectra in (b) show an as-deposited Ni-Fe film (dark yellow), and an empty graphene electrode (dark blue). **(c)** selected EDS spectra demonstrating Ni-rich and Fe-rich areas in the as-deposited Ni-Fe catalyst film. The areas with compositional inhomogeneity were also visible post-OER (not shown here). **(d)** The corresponding SEM image of the respective Ni-rich and Fe-rich areas shown in **(c)**. **(c)** and **(d)** are reproduced from previous work[1] under the terms of the CC BY Open Access license, which permits the reuse of our materials.

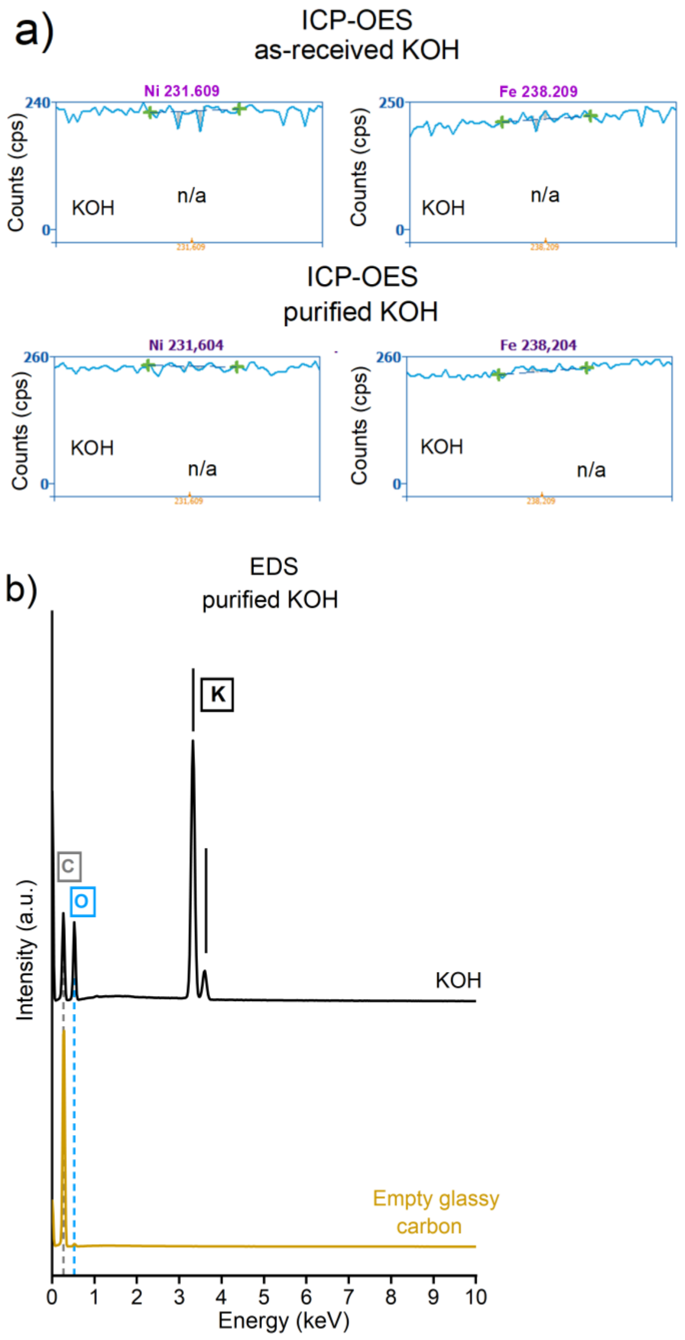

Figure S18. ICP-OES of the alkali hydroxide electrolytes addressing impurities. Only the relevant spectra are shown. The electrolyte had been diluted to a concentration of ~1000 ppm with respect to the cation (not shown here). **(a)** as-received alkali hydroxides and **(b)** EDS spectra recorded of the 1 M purified hydroxides dried on H_2SO_4 cleaned glassy carbon plates (4 x 4 mm, SIGRADUR® K, HTW) using the build-in detector in the Zeiss LEO1550 microscope to cross-check for other impurities. Elemental maps were recorded for ~10-15 min at 2-3 different areas (300 x 200 µm spot size) for each electrode, using an acceleration voltage of 15 kV. The variations in the carbon content in the EDS spectra depends on how homogenous the distributions of the dried salts are, and has no scientific significance. We do also not report the O-content since the accuracy is low for soft elements, but was on average 66 ± 9 at. % for the hydroxides, and ~3 at. % for the empty glassy carbon plate

Table S6. ICP-OES and EDS analysis of the Ni_xFe_{x-1} catalysts before and after OER in 0.1 M KOH electrolyte. The elemental compositions were determined using energy dispersive X-ray spectroscopy (EDS) and ICP-OES after the in situ XAS measurements and Tafel slope collection. The films were rinsed either briefly or more carefully with Milli-Q water in a more controlled rinsing step. All elemental compositions are reported in atomic %, and the metal loadings in weight %. The numbers reported in this table are the averages of several investigated areas or films.

**Material analyzed**	**After characterized in electrolyte:**	**Method**	**Films analyzed after:**	**Rinsing protocol**	**Elemental composition (at. %)**						**Metal loading (μg cm^{-2})**	
**EDS analysis (local)**					**Ni-rich area (at. %)**			**Fe-rich area (at %)**				
					**Ni**	**Fe**	**K**	**Ni**	**Fe**	**K**	**Ni**	**Fe**
Ni_xFe_{x-1}	as-deposited	EDS	n/a[(a)]	n/a[(a)]	66	34	0	34	66	0	n/a[(a)]	n/a[(a)]
Ni_xFe_{x-1}	KOH	EDS	XAS	Brief [(b)]	60	29	11	35	43	22	n/a[(a)]	n/a[(a)]
Ni_xFe_{x-1}	KOH	EDS	Tafel	Extensive [(c)]	71	29	0	n/a[(d)]	n/a[(d)]	n/a[(d)]	n/a[(a)]	n/a[(a)]
**ICP-OES analysis (bulk)**					**Bulk (at. %)**			**Bulk (at. %)**			**Bulk**	
					**Ni**	**Fe**	**X**	**Ni:Fe**			**Ni**	**Fe**
Ni_xFe_{x-1}	KOH	ICP-OES	XAS	Brief [(a)]	61	36	2	62:38			17	10

[(a)] does not apply
[(b)] The rinsing protocol was not carried out in a controlled manner, so either the films were rinsed briefly or not washed at all before analyzed.
[(c)] The samples were rinsed in a controlled manner, where the films were dipped five times in fresh Milli-Q water, and afterwards dried off with N_2 gas before analyzed.
[(d)] not investigated

S9. References

(1) Görlin, M.; Halldin Stenlid, J.; Koroidov, S.; Wang, H.-Y.; Börner, M.; Shipilin, M.; Kalinko, A.; Murzin, V.; Safonova, O. V.; Nachtegaal, M.; Uheida, A.; Dutta, J.; Bauer, M.; Nilsson, A.; Diaz-Morales, O. Key Activity Descriptors of Nickel-Iron Oxygen Evolution Electrocatalysts in the Presence of Alkali Metal Cations. *Nat Commun* **2020**, *11* (1), 6181. https://doi.org/10.1038/s41467-020-19729-2.

(2) Friebel, D.; Louie, M. W.; Bajdich, M.; Sanwald, K. E.; Cai, Y.; Wise, A. M.; Cheng, M.-J.; Sokaras, D.; Weng, T.-C.; Alonso-Mori, R.; Davis, R. C.; Bargar, J. R.; Nørskov, J. K.; Nilsson, A.; Bell, A. T. Identification of Highly Active Fe Sites in (Ni,Fe)OOH for Electrocatalytic Water Splitting. *J. Am. Chem. Soc.* **2015**, *137* (3), 1305–1313. https://doi.org/10.1021/ja511559d.

(3) Klaus, S.; Louie, M. W.; Trotochaud, L.; Bell, A. T. Role of Catalyst Preparation on the Electrocatalytic Activity of Ni1–xFexOOH for the Oxygen Evolution Reaction. *J. Phys. Chem. C* **2015**, *119* (32), 18303–18316. https://doi.org/10.1021/acs.jpcc.5b04776.

(4) Caliebe, W. A.; Murzin, V.; Kalinko, A.; Görlitz, M. High-Flux XAFS-Beamline P64 at PETRA III. *AIP Conference Proceedings* **2019**, *2054* (1), 060031. https://doi.org/10.1063/1.5084662.

(5) Kalinko, A.; Caliebe, W. A.; Schoch, R.; Bauer, M. A von Hamos-Type Hard X-Ray Spectrometer at the PETRA III Beamline P64. *Journal of Synchrotron Radiation* **2020**, *27* (1), 31–36. https://doi.org/10.1107/S1600577519013638.

(6) Dionigi, F.; Zeng, Z.; Sinev, I.; Merzdorf, T.; Deshpande, S.; Lopez, M. B.; Kunze, S.; Zegkinoglou, I.; Sarodnik, H.; Fan, D.; Bergmann, A.; Drnec, J.; Araujo, J. F. de; Gliech, M.; Teschner, D.; Zhu, J.; Li, W.-X.; Greeley, J.; Cuenya, B. R.; Strasser, P. In-Situ Structure and Catalytic Mechanism of NiFe and CoFe Layered Double Hydroxides during Oxygen Evolution. *Nat Commun* **2020**, *11* (1), 2522. https://doi.org/10.1038/s41467-020-16237-1.

(7) Zhao, Z.; Schlexer Lamoureux, P.; Kulkarni, A.; Bajdich, M. Trends in Oxygen Electrocatalysis of 3 D-Layered (Oxy)(Hydro)Oxides. *ChemCatChem* **2019**, *11* (15), 3423–3431. https://doi.org/10.1002/cctc.201900846.

(8) Zaffran, J.; Stevens, M. B.; Trang, C. D. M.; Nagli, M.; Shehadeh, M.; Boettcher, S. W.; Caspary Toroker, M. Influence of Electrolyte Cations on Ni(Fe)OOH Catalyzed Oxygen Evolution Reaction. *Chem. Mater.* **2017**, *29* (11), 4761–4767. https://doi.org/10.1021/acs.chemmater.7b00517.

(9) Winther, K. T.; Hoffmann, M. J.; Boes, J. R.; Mamun, O.; Bajdich, M.; Bligaard, T. Catalysis-Hub.Org, an Open Electronic Structure Database for Surface Reactions. *Sci. Data.* **2019**, *6* (1), 75. https://doi.org/10.1038/s41597-019-0081-y.

(10) Kresse, G.; Furthmüller, J. Efficient Iterative Schemes for Ab Initio Total-Energy Calculations Using a Plane-Wave Basis Set. *Phys. Rev. B* **1996**, *54* (16), 11169–11186. https://doi.org/10.1103/PhysRevB.54.11169.

(11) Kresse, G.; Hafner, J. Ab Initio Molecular Dynamics for Liquid Metals. *Phys. Rev. B* **1993**, *47* (1), 558–561. https://doi.org/10.1103/PhysRevB.47.558.

(12) Perdew, J. P.; Burke, K.; Ernzerhof, M. Generalized Gradient Approximation Made Simple. *Phys. Rev. Lett.* **1996**, *77* (18), 3865–3868. https://doi.org/10.1103/PhysRevLett.77.3865.

(13) Dudarev, S. L.; Liechtenstein, A. I.; Castell, M. R.; Briggs, G. A. D.; Sutton, A. P. Surface States on NiO (100) and the Origin of the Contrast Reversal in Atomically Resolved Scanning Tunneling Microscope Images. *Phys. Rev. B* **1997**, *56* (8), 4900–4908. https://doi.org/10.1103/PhysRevB.56.4900.

(14) Rao, K. K.; Zhou, L.; Lai, Y.; Richter, M. H.; Li, X.; Lu, Y.; Yano, J.; Gregoire, J. M.; Bajdich, M. Resolving Atomistic Structure and Oxygen Evolution Activity in Nickel Antimonates. *J. Mater. Chem. A* **2023**, *11* (10), 5166–5178. https://doi.org/10.1039/D2TA08854A.

(15) Blöchl, P. E. Projector Augmented-Wave Method. *Phys. Rev. B* **1994**, *50* (24), 17953–17979. https://doi.org/10.1103/PhysRevB.50.17953.

(16) Kresse, G.; Joubert, D. From Ultrasoft Pseudopotentials to the Projector Augmented-Wave Method. *Phys. Rev. B* **1999**, *59* (3), 1758–1775. https://doi.org/10.1103/PhysRevB.59.1758.

(17) Nørskov, J. K.; Rossmeisl, J.; Logadottir, A.; Lindqvist, L.; Kitchin, J. R.; Bligaard, T.; Jónsson, H. Origin of the Overpotential for Oxygen Reduction at a Fuel-Cell Cathode. *J. Phys. Chem. B* **2004**, *108* (46), 17886–17892. https://doi.org/10.1021/jp047349j.
(18) Gilmore, K.; Vinson, J.; Shirley, E. L.; Prendergast, D.; Pemmaraju, C. D.; Kas, J. J.; Vila, F. D.; Rehr, J. J. Efficient Implementation of Core-Excitation Bethe–Salpeter Equation Calculations. *Computer Physics Communications* **2015**, *197*, 109–117. https://doi.org/10.1016/j.cpc.2015.08.014.
(19) Vinson, J.; Rehr, J. J.; Kas, J. J.; Shirley, E. L. Bethe-Salpeter Equation Calculations of Core Excitation Spectra. *Phys. Rev. B* **2011**, *83* (11), 115106. https://doi.org/10.1103/PhysRevB.83.115106.
(20) Giannozzi, P.; Baroni, S.; Bonini, N.; Calandra, M.; Car, R.; Cavazzoni, C.; Ceresoli, D.; Chiarotti, G. L.; Cococcioni, M.; Dabo, I.; Corso, A. D.; Gironcoli, S. de; Fabris, S.; Fratesi, G.; Gebauer, R.; Gerstmann, U.; Gougoussis, C.; Kokalj, A.; Lazzeri, M.; Martin-Samos, L.; Marzari, N.; Mauri, F.; Mazzarello, R.; Paolini, S.; Pasquarello, A.; Paulatto, L.; Sbraccia, C.; Scandolo, S.; Sclauzero, G.; Seitsonen, A. P.; Smogunov, A.; Umari, P.; Wentzcovitch, R. M. QUANTUM ESPRESSO: A Modular and Open-Source Software Project for Quantum Simulations of Materials. *J. Phys.: Condens. Matter* **2009**, *21* (39), 395502. https://doi.org/10.1088/0953-8984/21/39/395502.
(21) Gonze, X.; Beuken, J.-M.; Caracas, R.; Detraux, F.; Fuchs, M.; Rignanese, G.-M.; Sindic, L.; Verstraete, M.; Zerah, G.; Jollet, F.; Torrent, M.; Roy, A.; Mikami, M.; Ghosez, Ph.; Raty, J.-Y.; Allan, D. C. First-Principles Computation of Material Properties: The ABINIT Software Project. *Computational Materials Science* **2002**, *25* (3), 478–492. https://doi.org/10.1016/S0927-0256(02)00325-7.
(22) Juhin, A.; de Groot, F.; Vankó, G.; Calandra, M.; Brouder, C. Angular Dependence of Core Hole Screening in $LiCoO_2$: A DFT+U Calculation of the Oxygen and Cobalt K-Edge X-Ray Absorption Spectra. *Phys. Rev. B* **2010**, *81* (11), 115115. https://doi.org/10.1103/PhysRevB.81.115115.
(23) Rehr, J. J.; Kas, J. J.; Vila, F. D.; Prange, M. P.; Jorissen, K. Parameter-Free Calculations of X-Ray Spectra with FEFF9. *Phys. Chem. Chem. Phys.* **2010**, *12* (21), 5503–5513. https://doi.org/10.1039/B926434E.
(24) Glatzel, P.; Alonso-Mori, R.; Sokaras, D. Hard X-Ray Photon-in/Photon-out Spectroscopy: Instrumentation, Theory and Applications. In *X-Ray Absorption and X-Ray Emission Spectroscopy*; John Wiley & Sons, Ltd, 2016; pp 125–153. https://doi.org/10.1002/9781118844243.ch6.
(25) Lafuerza, S.; Carlantuono, A.; Retegan, M.; Glatzel, P. Chemical Sensitivity of Kβ and Kα X-Ray Emission from a Systematic Investigation of Iron Compounds. *Inorganic Chemistry* **2020**, *59* (17), 12518–12535. https://doi.org/10.1021/acs.inorgchem.0c01620.
(26) Vankó, G.; Neisius, T.; Molnár, G.; Renz, F.; Kárpáti, S.; Shukla, A.; de Groot, F. M. F. Probing the 3d Spin Momentum with X-Ray Emission Spectroscopy: The Case of Molecular-Spin Transitions. *J. Phys. Chem. B* **2006**, *110* (24), 11647–11653. https://doi.org/10.1021/jp0615961.
